# Effect of Organic Ions on The Formation and Collapse of Nanometric Bubbles in Ionic Liquid/Water Solutions: A Molecular Dynamics Study

**DOI:** 10.1021/acs.jpcb.2c07950

**Published:** 2023-02-14

**Authors:** Raffaela Cabriolu, Bruno G. Pollet, Pietro Ballone

**Affiliations:** † Department of Physics, Norwegian University of Science and Technology (NTNU), 7491 Trondheim, Norway; ‡ Green Hydrogen Laboratory, Université du Québec á Trois-Riviéres, 3351 Boulevard des Forges, Trois-Riviéres, Quebec G9A 5H7, Canada; § School of Physics, University College, Dublin D04 V1W8, Ireland; ∥ Conway Institute for Biomolecular and Biomedical Research, University College, Dublin D04 V1W8, Ireland

## Abstract

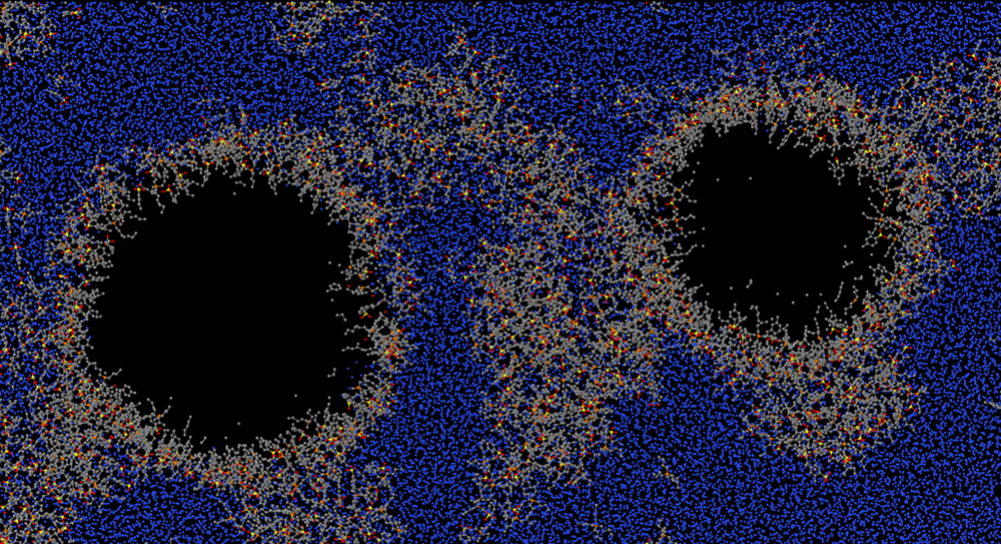

Molecular dynamics simulation is applied to investigate the effect of two ionic liquids (IL) on the nucleation and growth of (nano)cavities in water under tension and on the cavities’ collapse following the release of tension. Simulations of the same phenomena in two pure water samples of different sizes are carried out for comparison. The first IL, i.e., tetra-ethylammonium mesylate ([Tea][Ms]), is relatively hydrophilic and its addition to water at 25 wt % concentration decreases its tendency to nucleate cavities. Apart from quantitative details, cavity formation and collapse are similar to those taking place in water and qualitatively follow the Rayleigh–Plesset (RP) equation. The second IL, i.e., tetrabutyl phosphonium 2,4-dimethylbenzenesulfonate ([P_4444_][DMBS]), is amphiphilic and forms nanostructured solutions with water. At 25 wt % concentrations, [P_4444_][DMBS] favors the nucleation of bubbles that tend to form at the interface between water-rich and IL-rich domains. Cavity collapse in [P_4444_][DMBS]/water solutions are greatly hindered by a shell of ions decorating the interface between the solution and the vapor phase. A similar effect is observed for the equilibration of a population of bubbles of different sizes. The drastic slowing down of the bubbles’ relaxation processes suggests ways to produce long-lived nanometric cavities in the liquid phase that could be useful for nanotechnology and drug delivery.

## I Introduction

Longitudinal pressure waves propagating in a liquid at ultrasonic frequencies give origin to a variety of effects mediated by vapor bubbles that nucleate, grow, and interact during the expansion stage of the wave oscillation and eventually collapse when the pressure turns from negative to positive. The first effects of cavitation that have been discovered and investigated were mainly adverse, consisting of the corrosion of metal devices and the efficiency loss of nautical propellers.^1^ Cavitation itself has been the subject of documented scientific investigations and engineering since the 18th (Euler, ref (2)) and especially the 19th (Reynolds, ref (3)) centuries.

The nucleation and further evolution of bubbles in a liquid is a fundamental process that, besides natural phenomena and traditional technology, concerns a variety of aspects in food science,^4,5^ medicine,^6,7^ bio- and nanotechnology, and heat transport in microelectronics.^8^ In the form of ultrasonics, it enters essential laboratory protocols in chemistry and biology and provides the basis of sonochemistry.^9^ Increasingly, it is used in environmental applications.^10^ In many cases, cavitation occurs and has been investigated in water and in solutions of relatively simple solutes. Cavitation of solutions containing complex organic and ionic species^11^ might become increasingly relevant in advanced sonochemistry applications or might represent a secondary, perhaps unwanted effect for a wider variety of systems and processes. The ultrasonic frequency range used for medical therapy, diagnostics, and purposes of processing applications and sonochemistry research reaches up to 20 MHz,^9,12,13^ corresponding to a period of 50 ns.

From a conceptual point of view, the evolution of cavities in a fluid environment can be analyzed into their nucleation, growth, and collapse. This last stage might give origin to energetic events, manifested in sonoluminescence,^14^ due to the formation of a high-temperature plasma inside a collapsing bubble. A special case of experimental relevance is represented by the periodic oscillation of relatively long-lived bubbles, driven again by the propagation of an ultrasound wave.^11^

Bubbles’ conditions and dynamics have been investigated via kinetic and spectroscopic methods.^15^ Kinetic methods, such as comparative rate thermometry and methyl radical recombination techniques, in particular, are used to measure the temperature inside bubbles. The specific kinetic pathways determined with those approaches allow for temperature determination via the Arrhenius relation.^16−18^ Spectroscopic methods are also very popular for temperature and pressure determinations. In particular, the two-line radiance ratio and the wavelength shifts of emissions line protocols provide measures and also allow the control of emission temperatures as a function of driving acoustic pressure.^15^ Through volume oscillations-experiments, bubble dynamics is measured by light scattering in confined and bulk conditions.^19^ Electrochemistry has been used as well to investigate cavitation.^20^

Since the first research performed on single bubble sonoluminiscence (SBSL),^21^ numerous works have greatly contributed to the understanding of the single bubble dynamics.^22,23^ An additional topic of indisputable importance is the study of mutual interactions between bubbles. For example, Matula et al.^24^ reported characteristic line spectra for multibubble sonoluminiscence (MBSL) in contrast with the typical continuous spectrum reported in SBSL experiments. These results, in turn, stimulated numerous studies in the multibubble field.^11,25,26^ A quantitative correlation between sonoluminescence, sonochemistry, and acoustic emission from ultrasonicated aqueous solutions with different concentrations of sodium dodecyl sulfate (SDS) has been established.^11^ It has also been found that the addition of SDS influences the intensity of mutual interaction between bubbles,^27^ eventually creating an important disturbance in the dynamics of cavitation bubbles. Sadighi-Bonabi et al.^26^ numerically investigated the dynamics of two interacting cavitation bubbles in sulfuric acid solutions, concluding that the viscosity of the acid solutions seriously affects the interaction force between bubbles. In addition, Liang et al.^11^ found that the mutual interaction between bubbles is severely influenced by the cavitation state which has an important effect on the density and size of the bubbles, and, could eventually determine the creation of larger bubbles by coalescence. The size difference between bubbles is responsible for a nonlinear disturbance, meaning that, despite oscillations, a clear periodic feature is observed when bubbles have similar dimensions, while a nonlinear influence and dominant role is played by very large bubbles. In general, the kinetics of spontaneous nucleation in stretched simple liquids have been experimentally investigated by focusing sound waves in the liquid for which the nucleation rates in homogeneous conditions were reported in the range of 10^18^–10^27^ s^–1^ m^–3^.^11,28,29^

On the other hand, molecular simulations have the advantage to provide a detailed atomistic description of the formation and collapse of the bubbles. High stretches were investigated with standard Monte Carlo (MC)^30^ and Molecular Dynamics (MD) simulations,^31^ reporting nucleation rates with pressure and temperature dependence not aligned with the theoretical predictions from the Classical Nucleation Theory (CNT),^32,33^ but in reasonable agreement with some experimental results. However, at moderate stretches/tension, the order of magnitude of the nucleation rates would require very time-consuming direct molecular dynamics simulations. Different varieties of MD simulations using accelerated sampling methods have been carried out for homogeneous systems, consisting of model LJ particles,^34,35^ or more detailed models of solvents. Other studies addressed heterogeneous bubble nucleation at solid/liquid interfaces^38^ or investigated the effect on cavitation of nanoparticles in solution.^39^ In general, simulation results are interpreted in terms of CNT, which, however, is seriously inadequate, unless one considers the size dependence of the surface energy,^40^ an extension that, strictly speaking, brings the approach beyond the original CNT.

Thermodynamic aspects can also be investigated using classical density functional theory. Following nucleation, the time evolution of spherical cavities is modeled by the so-called Rayleigh–Plesset (RP) equation,^41,42^ which is a continuum hydrodynamics equation that adopts a macroscopic point of view to describe the dynamics of bubbles, whose validity has been tested by numerous experiments.^43^ This type of validation, however, has some limitations, since experiments are technically very difficult at this scale due to the fragility of bubbles,^44^ and, in addition, bubbles with radii smaller than 10 nm cannot be probed with current experimental techniques. As a consequence, a number of equilibrium and nonequilibrium molecular dynamics (NEMD)^45^ simulations have been carried out to verify the RP equation for bubbles at the nanoscale. The results have been somewhat mixed since, besides positive validations,^46−49^ less favorable assessments were given for instance in ref (50), and the need for improvements was highlighted for instance in ref (51). Evolution of the Rayleigh–Plesset formulation lead to more complex equations,^41^ i.e., Keller–Miksis and Gilmore–Akulichev models, developed to explain the response of bubble radius and bubble walls in terms of ultrasonication frequency, pressure, and gas diffusion under high amplitude ultrasound experiments. As already stated, in this study we are considering bubble formation on a nanometer scale under ultrasonic frequency with a period much longer than the bubble relaxation and dynamics. Furthermore, at variance from the setup commonly used in sonoluminescence experiments, our samples do not include dissolved gas species. In this simpler context, the more complicated models give the same answer as the original Rayleigh–Plesset equation, then, we will only consider the RP model in our discussion.^52−57^

We extend these types of investigations to include solutions of organic salts belonging to the room temperature ionic liquids variety. Two compounds are considered, i.e., tetra-ethylammonium mesylate [TEA][Ms] and tetrabutyl phosphonium 2,4-dimethylbenzenesulfonate [P_4444_][DMBS], whose schematic structures are shown in Figure S1 of the Supporting Information (SI). The first one is relatively hydrophilic, giving origin to water solutions virtually homogeneous down to the molecular scale. The second one is more markedly amphiphilic, its water solutions are nanostructured at room temperature and, at 50–50 wt % composition, become phase-separated above a lower critical solution temperature (LCST) of about 40 °C. At the 25 wt % IL composition of the simulated samples, the phase separation occurs at a temperature above the water boiling point, but already at room temperature the nanostructuring of the system is apparent. The focus of our study is precisely to investigate the interplay of nanostructuring with the processes of cavity formation, equilibration, and collapse in the cavitation context.

In what follows, tension θ is used as synonymous with a negative pressure, and it is defined in the present context as θ = −*P*, with both *P* and θ being positive definite quantities, expressed in the same units, either bar or kbar. Moreover, to avoid repetitions, we will also use *bubble* and *cavity* as synonyms, but the most appropriate term might be bubble, since all cavities contain water vapor, although, because of low vapor pressure and microscopic sizes, vapor molecules within the cavities are rarely seen during our simulations.

## II Methods and Model

Ultrasonic waves propagating in a liquid exert a periodic sequence of compression and expansion cycles, that, above a threshold intensity, result in cavitation, followed by bubble growth and eventual collapse. In most cases, the frequency of ultrasound extends up to just a few 10^2^ kHz, with a period of oscillation of the order of several μs, which still represents a challenging time scale for the large systems (number of atoms *N* > 10^5^, grouped into *M* distinct molecules) required for a minimally realistic simulation. However, in the case of nanometric bubbles, crucial steps in the whole cavitation/ultrasonic process such as the formation and growth of a bubble following nucleation, or its collapse once the negative pressure turns into a positive one, take times of the order of 50–100 ps, much shorter than even a single period of the pressure oscillations. In practice, these crucial stages take place under virtually constant pressure. Moreover, the formation of cavities is preceded by long nucleation times, and is separated from the collapse by a much longer stage in which bubbles fluctuate, evolve slowly following the pressure variations, and interact with each other through the liquid medium. These considerations suggest that cavities’ formation, mutual interaction and collapse can be investigated separately, using molecular dynamics in the NPT ensemble. This is the primary approach used in the present study, accepting that the validity of the results might be negatively affected at sonication frequencies exceeding the MHz or when considering bubbles whose radius is of μm scale.

A slightly different protocol, however, is used to investigate the cavity nucleation stage, since the microscopic size of the simulation systems, together with the large overtension required to create a bubble on the ns time scale, imply that a bubble growing at NPT conditions would quickly break the continuity and connectivity of the simulated sample (as shortly described in Figure S2 of the SI), suppressing the fluid resistance to further expansion, and leading to the uncontrollable growth of the volume simulated in the NPT ensemble. This is not a problem in the determination of the mechanical stability range of a homogeneous fluid under tension carried out in section A, since in that case the cavity formation and the breaking of the sample represent the natural end of the simulation. However, the detailed investigation of nucleation and growth has been carried out in the NVT ensemble, in order to obtain a cavity that rapidly settles into a stationary state, that can be characterized and observed indefinitely.

To summarize these initial considerations on the simulation protocol: for reasons of computational expediency, we investigate the effect of ultrasound waves by analyzing separately three stages, consisting of (i) the nucleation and growth of bubbles in the systems at NVT conditions under a suitable tension θ (−θ = *P* ≪ 0); (ii) the evolution of interacting bubbles at nanometric separation, carried out again at NVT conditions; (iii) the collapse of single bubbles at constant positive pressure, simulated at isobaric-isoenthalpic (NPE) conditions, adopted in order to analyze the changes of potential energy and the distribution of kinetic energy among molecules unaffected by the ad-hoc dynamics of thermostats. In a preliminary stage, the mechanical stability limit of the stretched homogeneous liquid with respect to cavitation is determined by MD in the NPT ensemble.

As described in more detail in the following section, the samples consist of water, or of an organic ionic salt of the room temperature ionic liquid variety dissolved in water at 25 wt % concentration. Molecular dynamics simulations in the ensembles already listed are carried out based on an empirical atomistic force field. The [TEA][Ms] and [P_4444_][DMBS] ionic liquids are modeled by a Gromos^58^ force field (version 54A7).^59^ The force constants and charges needed to parametrize the IL force field are obtained from the ATB Web site.^60^ As detailed in ref (59), tuning the solubility of molecules in SPC water has been part of the force field development. Therefore, for consistency, the SPC model^61^ is used to simulate water.

All simulations are carried out using the Gromacs package,^62^ version 2019. Simulation conditions used in the different stages of the present study, including system size and composition, pressure, volume, temperature, time step, and total simulation time are given in the following section III.

The analysis of trajectories is the most crucial part of the simulation protocol. In the cavitation stage under tension, simulated at NVT conditions, the sudden change of pressure, becoming less negative, is used as the main diagnostic quantity to identify the formation of a stable mesoscopic cavity, of diameter ∼10 nm. In all cases, the analysis of trajectories aims primarily at identifying and quantifying every void in the system, either stable or flickering, determining its volume and shape. As it was done in previous studies,^35^ a set of representative configurations is analyzed, identifying all voids in each of them by inserting test particles not overlapping among themselves nor with real particles in the system, placing them at the nodes of an auxiliary lattice, or at random trial positions, using some kind of Monte Carlo approach. After a full sweep of the configuration, the set of inserted particles provides a negative image of the system, highlighting void spaces.

Although writing a code to implement this algorithm is trivial, for the sake of simplicity and also of reproducibility, we carry out the insertion of test particles (water molecules, in this case) using the solvate routine of Gromacs.^62^ The oxygen atoms of the water molecules added to the system are seen as the complement of the real system, thus representing voids. Because of this peculiar way of identifying voids, the size of cavities is first measured by the corresponding number *N* of ghost water molecules. This discrete measure is then converted into the cavity volume Ω measured in nm^3^. The conversion between the two is represented by *V*_*w*0_ = 0.03065 nm^3^, volume of a water molecule at equilibrium, estimated at ambient (*P*, *T*) conditions, hence, Ω = *V*_*w*0_*N*.

Following the insertion of void-particles, a population of distinct bubbles is identified by a homemade clustering algorithm, grouping the elementary voids into disjoint sets (clusters) consisting of void-particles separated among themselves by less than a cutoff distance equal, in our analysis, to *r*_Hbond_ = 3.2 Å. For the sake of simplicity, this analysis is based on the oxygen atoms added by the solvate utility, neglecting the corresponding hydrogens. The population of bubbles identified in this way and averaged over a representative set of configuration is characterized in terms of the size probability distribution and in terms of geometric properties for the single clusters of largest size. Most of the cavities identified by the added water molecules are small, having a volume of 0.3–0.5 nm^3^. At high tension, and/or long times, a single large bubble of a few to a few hundred nm^3^ nucleates and grows. Our simulations are carried out at NVT conditions. Therefore, after nucleation, the bubble will grow up to a stationary volume Ω. As discussed in the following sections and in the SI, Ω depends on the sample volume *V* and on the surface tension γ of water or of the IL/water solutions.

In addition to volume, the major property defined for the single cluster of ghost water molecules concerns its shape, which is characterized by its inertia tensor:
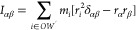
1where *i* ∈ *OW*′ indexes the oxygen atoms of the water molecules added by the solvate routine, {*r*_α_, α = 1, ..., 3} are Cartesian coordinates measured from the center of mass of the cluster, *r*_*i*_^2^ = **r**_**i**_·**r**_**i**_, and *m*_*i*_ is the mass of particle *i*. Since, in our study, the inertia tensor accounts for (ghost) oxygen atoms only, and it is introduced to characterize the system geometry and not also its rotational dynamics, we set *m*_*i*_ = 1 for all particles. Diagonalization of *I*_αβ_ gives the three principal momenta of inertia { ϵ_11_, ϵ_22_, ϵ_33_ } from which two parameters have been computed, i.e.:
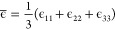
2and
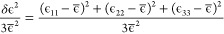
3The first parameter is an additional measure of the size of the cluster and can be translated into a gyration radius *R*_g_ using the relation ϵ̅ = *NR*_g_^2^, where *N* is the size of the cavity measured in *V*_*w*0_ units. The second parameter measures the deviation from sphericity.

The collapse of bubbles is investigated at NPE conditions, releasing the constraint on the system volume, and monitoring the shrinking of the bubble volume as well as the changes in the local temperature (kinetic energy). Since the temperature might increase and energetic events could occur during the late stages of collapse, the time step is reduced to 0.1 fs to ensure good energy conservation.

An analytical tool to characterize and predict the time dependence of the cavity volume is represented by the Rayleigh–Plesset (RP) equation. For a spherical bubble of volume Ω in a fluid of mass density *w*, surface tension γ, and viscosity η, the RP equation states that
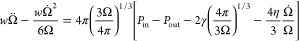
4where *P*_in_ and *P*_out_ denote the pressure inside and outside the bubble, respectively. At stationary conditions, Ω̈ = Ω̇ = 0, and eq 4 reduces to the static Laplace equation:
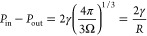
5where *R* = (4π /3Ω)^(1/3)^ is the radius of the cavity, assumed to be a sphere. The comparison with simulation results, however, requires a detailed consideration of the simulation conditions, such as the choice of the NPT or NVT ensemble, and the size of the simulated system as discussed below and in SI.

In many cavitation studies, a basic target of the investigation is the time to nucleate a cavity in the homogeneous liquid system under tension. This time is a random variable, whose probability distribution depends exponentially on the activation free energy for cavitation, and also depends on the sample volume, since the rate of cavity formation on the whole system is proportional to its volume. Since the simulated samples need to be large, and nucleation times tend to be long, in general one cannot perform a number of simulations sufficient to determine the full probability distribution with any meaningful error bar. Hence, for every system of interest, we determine by simulation the time τ_*f*_ of the first occurrence for the formation of a stable cavity that breaks the system homogeneity. Assuming that the probability distribution of cavitation is peaked at the most probable value, τ_*f*_ is a good approximation for the average cavity nucleation time. To compensate for the inherent volume dependence of the result, we carry simulations of cavity formation for pure water and IL/water samples of nearly equal size. In this way, the direct comparison of simulation results provides the trends relevant to the discussion. One additional water sample of larger size has been simulated as well to provide insight into the volume dependence of results.

## III Results

Simulations have been carried out for two samples consisting of organic ionic salts dissolved in water at ∼25 wt % concentration. The size of the two samples is set to (*N*_w_ = 64600, *N*_IL_ = 1920) for the [Tea][Ms]/water case, and (*N*_w_ = 65285, *N*_IL_ = 874) in the [P_4444_][DMBS]/water case, where *N*_w_ and *N*_IL_ are the number of water molecules and neutral ion pairs, respectively. For comparison, a water sample consisting of 84400 water molecules (Small Water sample) has been simulated as well. The number of atoms (*N* = 253200) and volume (*V* ∼ 2600 nm^3^) of this water sample are similar to those of the IL/water solution samples, ranging from 250 × 10^3^ to 260 × 10^3^ atoms, and *V* ∼ 2500 nm^3^. Simulations have been carried out also for a water sample consisting of *N*_w_ = 200000 water molecules (Large Water sample), aiming at investigating the size dependence of selected results.

Most of the simulations have been carried out at *T* = 300 K, but, again for comparison, simulations at *T* = 280 and 320 K have been carried out for the small water sample.

### A Stability Limit of Water Samples under Tension

The first task carried out by MD simulations is the determination of the stability limit of the water and IL/water samples under tension. In the case of water, this mainly represents a test of the method and a calibration of the analysis of trajectories. To this aim, we first considered the Small Water sample of *N*_w_ = 84400, equilibrated at NPT conditions over ∼100 ns during previous simulations at *T* = 300 K and *P* = 1 bar. The pressure parameter of the NPT barostat was decreased in discontinuous steps of 50 bar, the system briefly equilibrated during 100 ps always at NPT, and the procedure repeated until a bubble formed and the system, reducing its ability to withstand tension, thus driving the sample breaking while the internal virial nearly vanished. Since the breaking occurs during the 100 ps of the short local equilibration, we considered the last value of the barostat before cavitation as the limit of mechanical stability θ_crit_ under tension, at which the nucleation of the bubble is virtually barrierless. The way the failing of the sample under tension is detected is illustrated in Figure S3 of the SI. The same procedure, with the local equilibration time raised to 200 ps at each pressure step gives again virtually the same estimate of the stability limit, showing that this property does not depend much on the details of our definition.

**Figure 1:**
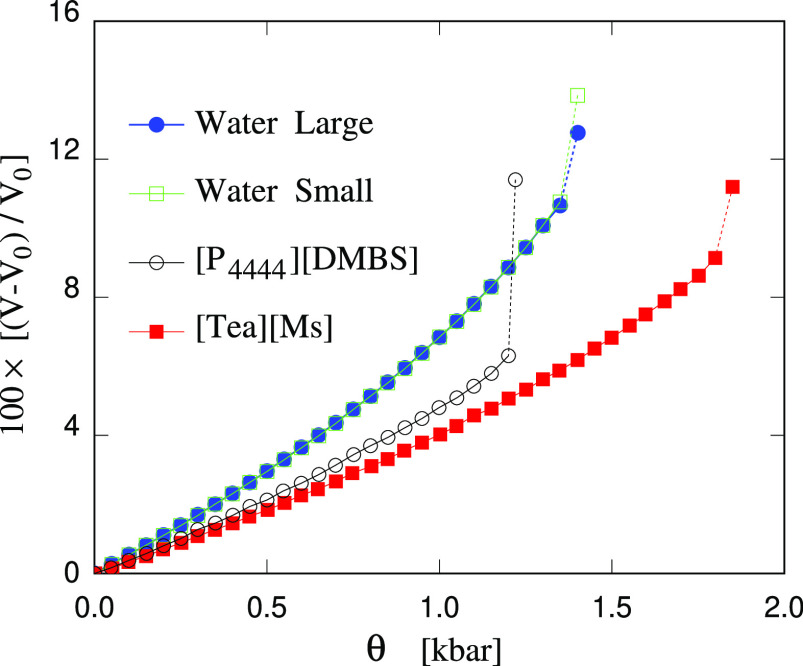
*V*(θ) phase diagram (where θ = −*P*) showing the stability range of homogeneous samples with respect to cavitation. Each point represents a NPT simulation at *T* = 300 K. The near discontinuity at the end of each curve marks the formation of a cavity that breaks the sample homogeneity. *V*_0_ is the equilibrium volume of each sample at *P* = 1 bar.

The results of this stage of our study are displayed in the form of the θ(*P*) phase diagram by the empty squares and green line in Figure 1, while the θ_crit_ values are summarized in Table 1. The same computational experiment carried out following the same protocol for the Large Water–water sample of *N*_w_ = 200000 molecules provides virtually the same result (see the full dots and blue line in Figure 1), breaking at the same pressure to within the 50 bar resolution of our negative pressure scan thus showing that already for *N*_w_ ≳ 80000 the nucleation barrier does not depend on the sample size. Moreover, the near coincidence of the breaking point shows that the vanishing of the activation barrier, which does not depend significantly on size, is the important factor determining the mechanical stability range, while the proportionality of overall nucleation rate and volume has little effect on the estimation of θ_crit_.

**Table 1:** Limiting Tension θ_crit_ Marking the Transition from Metastability to Mechanical Instability of Homogeneous Samples

	water large	water small	[tea][Ms]/water	[P_4444_][DMBS]/water
θ_crit_ [kbar]	1.40	1.40	1.80	1.25

The stability limit for water at θ_crit_ = 1400 bar estimated by the present simulation agrees with previous experimental estimates, giving from θ_crit_ = 1000 bar in ref (36) to θ_crit_ = 1400 bar in ref (37).

The mechanical stability range under tension can be tuned by the addition of suitable salts. As already stated, we considered water solutions of two organic salts of low melting temperature. The *V*(θ) (or, equivalently, *V*(*P*)) phase diagram reported in Figure 1 shows that both salts increase the bulk modulus *B* = −*V* d*P*/d*V* of the solution with respect to water, apparently because of the enhanced cohesion due to Coulomb interactions. The two salts, however, differ in their degree of hydrophobicity and surface activity. The first one, i.e., [Tea][Ms], is relatively soluble, while the second one, i.e., [P_4444_][DMBS], is more hydrophobic and surface active since it decreases the surface tension of its water solutions with respect to water. These differences are reflected in the mechanical stability range under tension, also shown in Figure 1. Addition of [Tea][Ms] at 25 wt % composition widens the stability range by about 400 bar. The effect of [P_4444_][DMBS] at the same concentration is somewhat weaker, and of the opposite sign, since it reduces the stability limit by about 150 bar, presumably because it decreases the surface tension of the bubbles, thus decreasing both the nucleation free energy and the size of the critical bubble.

As expected, decreasing temperature from *T* = 300 K to 280 K increases the stiffness as well as the surface tension of the sample and enhances its stability with respect to the increasing tension, while increasing temperature to *T* = 320 K has the opposite effect (see Figure S4 in the SI). The change of temperature by 20 K on either side of *T* = 300 K changes the stability limit by about 200 bar. As briefly discussed in the SI on the basis of the RP equation, the cavitation property of a fluid are primarily determined by its bulk modules *B* and surface tension γ. Because of the monotonic dependence of *B* on *T*, the effect of temperature on the mechanical stability under tension is not exactly that of dissolving salts. However, this test shows that the effect of dissolving organic salts at 25 wt % concentration is comparable to that of changing *T* by 20–30 K. Moreover, it suggests that the most important effect on stability with respect to nucleation is due to the surface activity of the dissolved electrolyte, while the bulk modulus *B* plays a less dominant role.

### B Cavity Formation in Water Samples

At tension θ below the stability limit θ_crit_, the formation of a bubble is opposed by a free energy barrier and requires a nucleation time that increases rapidly with decreasing tension. As discussed in section II, the formation of bubbles in this metastable regime has been investigated by simulations in the NVT ensemble, thus preventing the complete breaking and unrestrained expansion of the sample following the cavity formation. These simulations started from the configurations stored at the end of the short NPT equilibration runs carried out while ramping θ up to the stability limit θ_crit_ in steps of 50 bar. For all systems we considered, i.e., the two water and the two IL/water samples, the formation of a bubble at NVT conditions has been observed within a relatively narrow range (∼150 bar) of negative pressures close to θ_crit_. At lower tension, the observation of bubble formation might have been hampered by the limited duration of the simulation runs (up to 600 ns), but in small samples at NVT condition, bubble formation might be prevented altogether below a minimum tensionθ̅ of the order of a few 10^2^ bar, as discussed below and in the SI.

**Figure 2:**
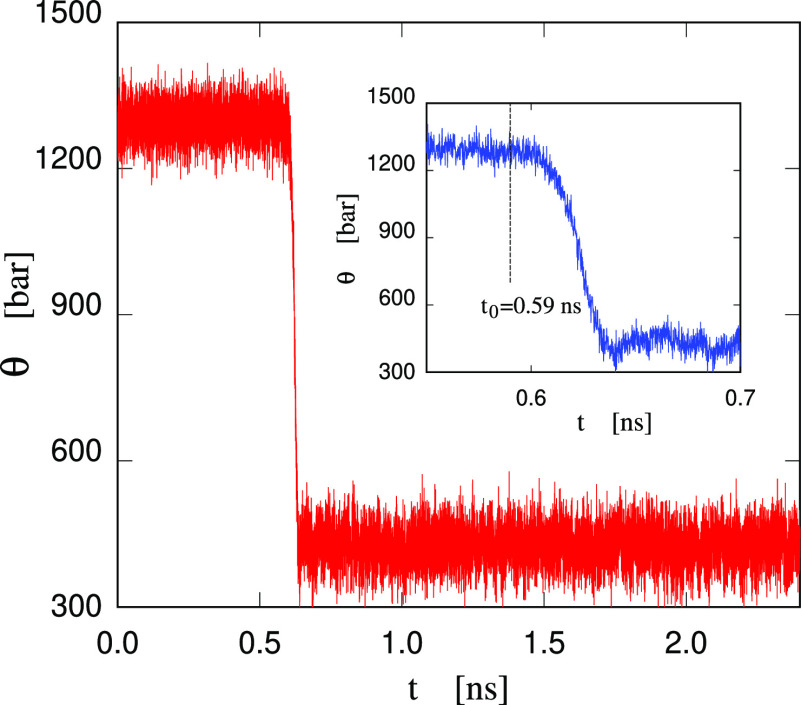
Tension θ (= −*P*) in the small water sample as a function of time during an NVT run at the volume of the homogeneous state at θ = 1300 bar, *T* = 300 K. The release of tension starting at *t*_0_ = 0.59 ns (see the dashed line in the inset) marks the nucleation of a stable bubble. The inset giving an expanded view of the formation process shows that pressure stabilizes ∼40 ps after nucleation, reaching an average value of θ = 425 bar.

**Figure 3:**
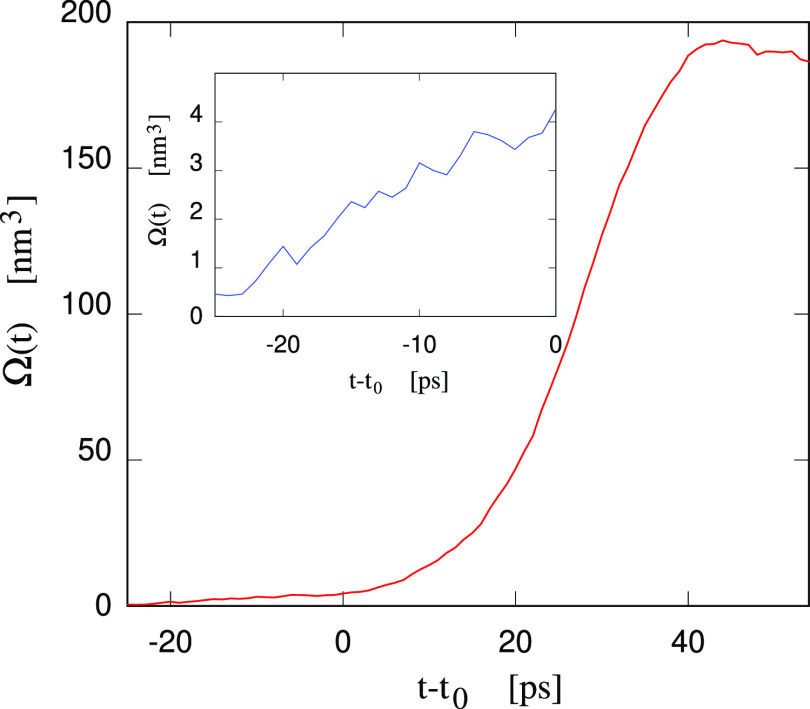
Volume of the largest void in the Small Water sample at cavity nucleation. NVT simulation at the sample volume *V* = 2847 nm^3^ of the homogeneous system at θ = 1300 bar, *T* = 300 K. The origin of the time scale corresponds to the beginning of nucleation, identified by the vertical dashed line in the inset of Figure 2. The inset gives an expanded view of the same quantity at *t* < 0.

**Figure 4:**
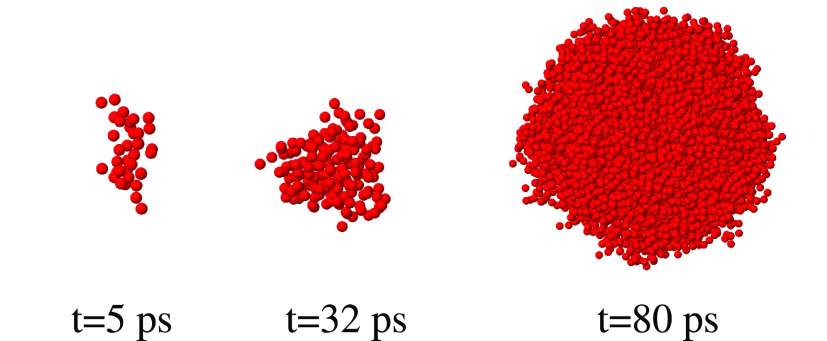
Snapshots of the largest void in the small water sample at three different times. NVT simulation at the volume of the homogeneous sample at *P* = −1300 bar, *T* = 300 K. The origin of the time scale corresponds to the beginning of nucleation, identified by the vertical dashed line in the inset of Figure 2.

Let us consider the case of the Small Water sample (*N*_w_ = 84400) as an example, simulated at the volume *V*_start_ = 2847 nm^3^ corresponding to θ = 1300 bar in the homogeneous state. As shown in Figure 2, the fluctuating negative pressure suddenly starts to rise, marking the nucleation of a stable bubble. Pressure in the fluid and size of the bubble increase rapidly during a growth stage lasting ∼40 ps (see Figure 3), then reaching a stable condition in which the pressure in the sample and the bubble size oscillates around θ = 425 ± 4 bar and Ω = 190 ± 1 nm^3^, respectively. A slight overshoot is apparent in both the system pressure and size of the cavity at the end of the formation stage. Moreover, if we take the first detectable increase of pressure at *t* = *t*_0_ as the beginning of nucleation, then the analysis of the largest void in the sample reveals that nucleation arises from an event taking place ∼20 ps earlier, as can be appreciated in Figure 3. In other terms, monitoring the size of the largest cavity in the sample is a more sensitive indicator of bubble nucleation. However, we keep our primary definition of nucleation time based on the pressure change, because this can be read directly from the simulation output, while the determination of the size of all cavities requires additional analysis. A few snapshots documenting the growth of the bubble are shown in Figure 4, while a more detailed sequence is in SI, consisting of a series of 50 snapshots taken every picosecond starting from nucleation. It is apparent that the cavity has a somewhat irregular shape at the beginning of its formation, and becomes increasingly isotropic with increasing size and with the stabilization of pressure following the bubble formation. This aspect of the cavity geometry is quantified by computing the principal momenta of inertia, determined from the number and configuration of the water molecules added by the algorithm described in section II The results are shown in Figure S5 of the SI and qualitatively agree with those of previous studies (see, for instance, ref (40)).

The cavity formed during the NVT simulation, once it reaches equilibrium, accounts for a major fraction of the excess volume (*V* – *V*_0_) resulting from expanding the sample. This fraction, moreover, increases monotonically, although slowly, with increasing (*V* – *V*_0_). In water, this fraction reaches 90% at the volume of the mechanical stability/instability transition for the homogeneous system. The remaining excess volume fraction, not released by the cavity, is responsible for the residual tension θ_res_ in the sample. Moreover, as expected, in any given sample the size of the equilibrium cavity decreases with decreasing tension in the starting homogeneous system (see Table 2). As apparent again from the data in Table 2 and contrary to intuition, in each sample the residual tension at the end of the cavity formation at NVT conditions, increases with decreasing cavity volume. Moreover, a comparison of the results for the Small and Large Water samples shows that at equal initial tension θ_start_ the size of the cavity grows somewhat more than linearly with increasing sample size. Some of these effects are caused or amplified by the small size of the simulated samples, as briefly discussed in the SI starting at page 17.

**Table 2:** Parameters Characterizing the Nucleation and Stationary State of a Bubble at NVT Conditions^a^

sample	θ_start_ (bar)	*V*_start_ (nm^3^)	τ_*f*_ (ns)	⟨Ω⟩ (nm^3^)	θ_res_ (bar)
Small Water	0	2588			
	1400	3102	0.02	451.8	333
	1350	2865	0.12	209.0	416
	1300	2847	0.59	190.0	425
	1250	2832	23.09	172.3	436
large water	0	6130			
	1400	6854	0.016	592.3	340
	1350	6788	0.28	523.1	348
	1300	6740	3.98	477.8	358
	1250	6715	7.62	442.9	363
[tea][Ms]/water	0	2418			
	1800	2639	0.05	164.7	546
	1750	2627	0.48	154.3	568
	1700	2617	9.65	142.2	583
[P_4444_][DMBS]/water	0	2488			
	1250	2766	0.011	214.0	499
	1200	2645	0.69	82.1	670
	1150	2626	5.86	61.2	678

a All simulations start from a metastable homogeneous sample (no bubble) briefly equilibrated at NPT conditions during 100 ps at θ = θ_start_ and *T* = 300 K. The volume *V*_start_ of the homogeneous sample estimated during the NPT run is adopted for the NVT run. τ_*f*_ is the time elapsed before a stable bubble is first observed, ⟨Ω⟩ is the average volume of the bubble at equilibrium and θ_res_ (= −*P*_res_) is the residual average tension after equilibration following the bubble formation. Each system has been simulated once, hence the uncertainty on the time τ_*f*_ cannot be estimated. For each sample, the highest θ_start_ is equal to the critical θ_crit_ of Table 1. The lowest θ_start_ > 0 that has been simulated in view of cavitation is limited by the requirement that nucleation of the bubble occurs within a few 10^2^ ns, i.e., τ_*f*_ < a few 10^2^ ns. The error bar on ⟨Ω⟩ is about 0.6 nm^3^. The error on θ_res_ is about 2 bar.

**Figure 5:**
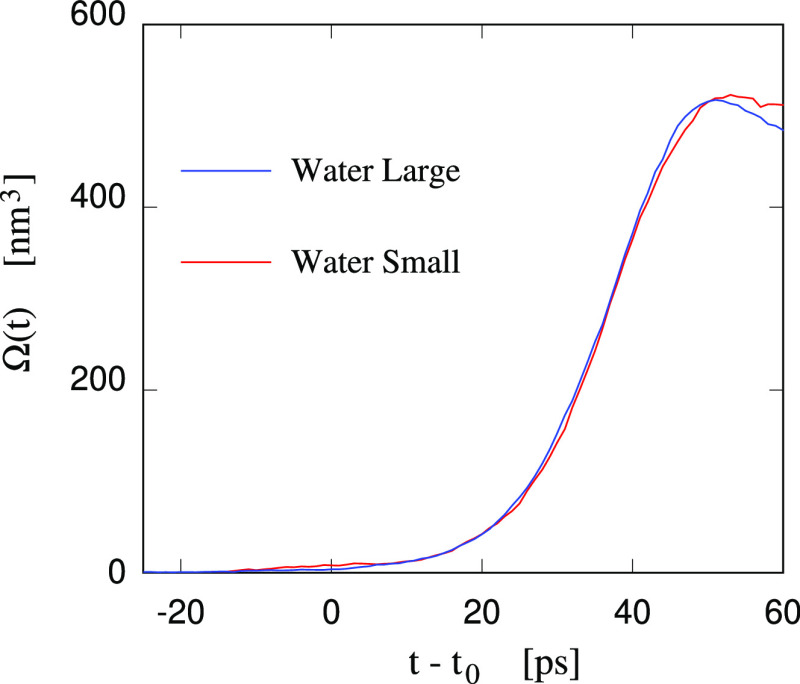
Time dependence of the cavity volume Ω in the Small and Large Water samples whose tension before the bubble formation is θ = 1300 bar. The time *t* of the two simulations has been shifted to have the same nucleation time *t*_0_ in both samples. The cavity size in the small sample has been multiplied by the constant factor *k* = 2.515 corresponding to the ratio of the equilibrium cavity size in the two samples.

Comparison with the results of NVT simulations carried out at different values of the starting tension θ_start_ < θ_crit_ shows that, in each sample, the duration of the cavity growth stage depends moderately on the starting tension, increasing slowly with decreasing θ_start_, despite the decrease of the cavity volume to be cleared of water. Moreover, the comparison of the data for the Small and Large Water samples shows that starting from the same tension, θ_start_, the growth of the cavity takes the same time irrespective of the sample size, despite a sizable difference in the volume of the two resulting cavities. In fact, after shifting the origin of time in order to have the same nucleation, *t*_0_, it is possible to virtually superimpose the two plots of the cavity size as a function of time simply by multiplying size by a constant factor (see Figure 5) equal to the ratio of the volume of the two cavities at equilibrium in the two samples. This observation can be rationalized in terms of the RP equation, as already reported in the literature.^9,49^ The result implies that during growth (which occurs under a sizable tension) the water/vapor interface moves at a speed that is linearly proportional to the radius of the equilibrium cavity. Interpolating the data of Figure 5, the expansion of the cavity in the small water sample (*R* ∼ 2.5 nm) reaches 50 m/s at *t* = 36 ps after *t*_0_, while it reaches 100 m/s at the corresponding time in the Large Water sample (*R* ∼ 5 nm). Both values are far below the sound velocity in water (*v*_s_ = 1480 m/s), which however would be reached in the formation of a cavity with *R* ∼ 0.1 μm if the linear relation of expansion rate and cavity radius holds over a few orders of magnitude. Further computations with significantly larger samples are needed to determine the validity range of the linear relation between radius and expansion velocity during the cavity formation. Even assuming a progressive deviation below the linear scaling, it is likely that cavities of μm radius (or more) will form at sonic speeds. As shown below, in water (and, we anticipate, in [Tea][Ms]/water solutions) the collapse of bubbles takes place during times similar to those of formation and with a similar linear scaling with the cavity radius. Therefore, the collapse of μm bubbles will also generate shock waves that will play a major role in sonochemistry and sonoluminescence.

A crucial question of cavitation concerns the size and properties of the critical cavity in a fluid under tension, which, by definition, has a 50% chance of growing into a stable bubble and a 50% chance of decaying spontaneously. This aspect is investigated in two complementary ways. The first analysis is based on the computation of the size distribution of unstable (small) cavities in homogeneous systems under tension simulated at NVT conditions. We consider homogeneous samples under tension θ such that the system will eventually develop a stable cavity, as, for instance, the large water sample at θ = 1300 bar. We limit our analysis to a portion of the trajectory corresponding to *t* ≪ *t*_0_, where, as before, *t*_0_ is the time of the stable bubble nucleation. In practice, we considered times *t* such that *t*_0_ – *t* > 1 ns. It is easy to verify that all the cavities identified in this way are unstable and do not evolve directly into a stable bubble. Therefore, the largest cavity found in this analysis could be considered as a lower bound for the critical cavity size at tension θ. This criterion is not rigorous, since it neglects the statistical nature of the critical cavity whose definition includes the 50% chance of growing and 50% chance of collapsing. However, together with other observations, this analysis gives us an idea of the size and properties of the critical nucleus.

**Figure 6:**
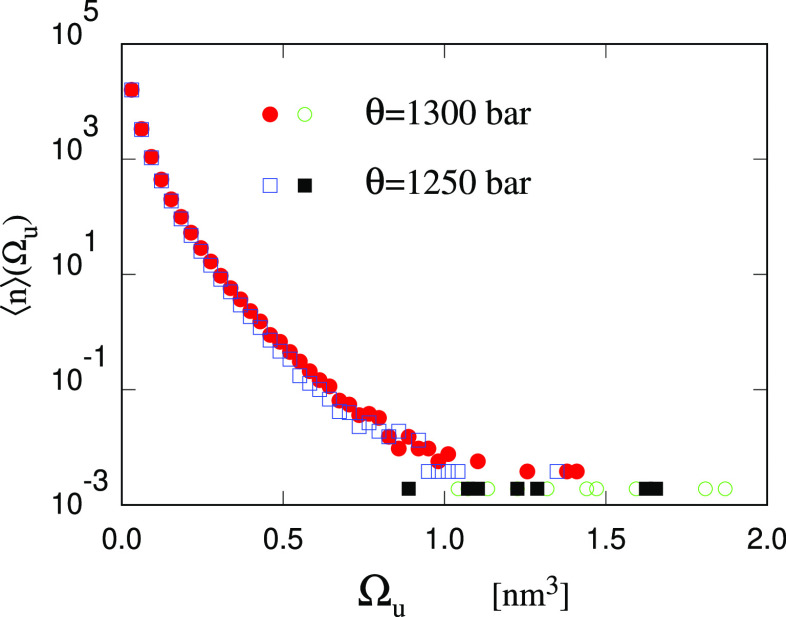
Average number ⟨*n*⟩ of unstable cavities in the Large Water sample as a function of cavity volume Ω_*u*_. Time average carried out on the homogeneous sample before the nucleation of a stable cavity, considering 500 configurations separated by 2 ps from each other. The red dots and the empty squares refer to sizes that have been seen multiple times during the simulation. The empty circles and the filled squares refer to sizes seen only once, for which the probability of growing/decaying has 100% error bar.

The results of this analysis are summarized by the average number ⟨*n*⟩(Ω_*u*_) of unstable cavities of size Ω_*u*_ observed in the system at any given time, reported in Figure 6 for the Large Water sample at θ = 1250 and 1300 bar. The distribution of sizes is peaked at the smallest sizes and covers a narrow interval, hence a logarithmic scale has been used to represent the simulation data. As apparent from the figure, at any given time, we observe thousands of cavities whose size is less than 0.6 nm^3^ (20*V*_*w*0_), but the analysis of hundreds of configurations is required to accumulate sufficient statistics for 0.6 ≤ Ω_*u*_ ≤ 1 nm^3^. The most interesting result of this analysis is that, although rarely, we observed near-nucleation events, or perhaps we should call them failed nucleation events, identified by a relatively long-lived (30–50 ps) cavity whose size, although still small, exceeds the background range by a factor of 3 or more, reaching up to 2.5 nm^3^, equivalent to 80*V*_*w*0_. None of these prenuclei evolved into the equilibrium bubble, whose volume is at least 50 times larger. An example of failed nucleation lasting 40 ps is given in the SI as a sequence of snapshots collected in an *xyz* trajectory file. Together with the data for the early stages of nucleation shown at beginning of this section, these results suggest that the critical cavity size in water is between 1.8 and 3.0 nm^3^, both at θ = 1300 and 1250 bar. As is also apparent from Figure 6, the dependence of the distribution on tension is moderate, at least up to the cavity volume Ω_*u*_ ∼ 1 nm^3^ for which sufficient statistics is achievable. At larger cavity volumes (Ω_*u*_ ≥ 1 nm^3^), the probability distribution for θ = 1300 bar, as expected, seems to be higher than the one for θ = 1250 bar, consistent with the fact that the free energy barrier for the cavity nucleation increases with decreasing θ. Statistics, however, is not sufficient to reach quantitative conclusions on this point.

**Figure 7:**
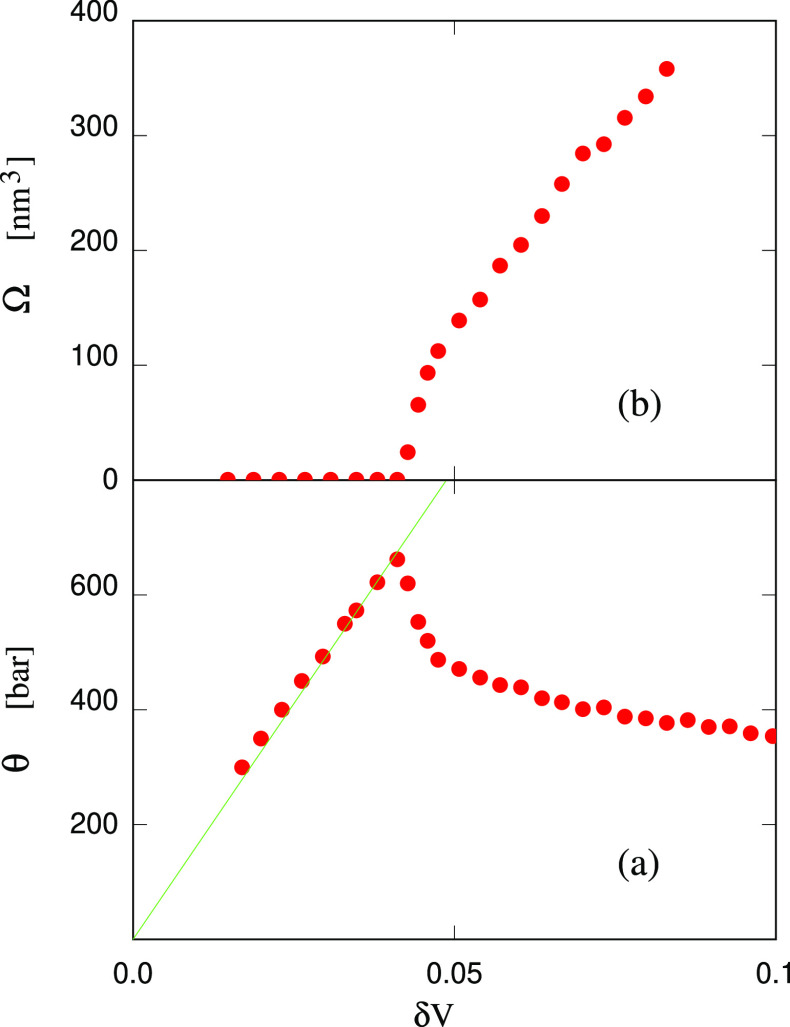
Tension in the large water sample and volume Ω of the stable cavity as a function of the sample volume *V* while squeezing the system with a bubble inside, starting from the volume such that the homogeneous systems has a tension θ_start_ = −*P*_start_ = 1300 bar. δ*V* = (*V* – *V*_0_)/*V*_0_, where *V*_0_ = 6130 nm^3^ is the equilibrium volume at *P* = 1 bar. Each dot represents a 200 ps NVT simulation carried out in sequence, moving from the right to the left of the figure. The straight dashed line is the elastic θ(*V*) relation in the stretched homogeneous sample.

A different way to analyze the properties of the critical cavity without replicating the long nucleation simulations, is to start from a sample in which a stable bubble has been formed and equilibrated, and then progressively reduce the system volume observing and characterizing smaller and smaller cavities until the system reverts to a homogeneous state. The smallest stable bubble observed in this way offers a glimpse of a critical cavity and of its properties. The picture obtained in this way cannot be superimposed to the one obtained from the stationary size distribution of Figure 6 because they are obtained under different conditions since in the previous case the volume is kept fixed, while in the present case the volume is progressively reduced. In some sense, this second method aims at the least critical among the critical nuclei, corresponding to the smallest sample volume and the largest nucleation barrier. As an example, we consider again the large water sample at the volume corresponding to θ = 1300 bar for the starting homogeneous system, in which a bubble has formed, reducing the tension to θ_res_, and has been equilibrated at NVT conditions during several ns. Then, the volume is progressively reduced in small steps of 0.3%, and the sample is equilibrated again for 200 ps following each variation in the volume. The results for the pressure in the system and the volume of the stable bubble are reported in Figure 7, whose similarity with Figure S19 of SI is apparent. As in the solution of the RP equation represented in Figure S19, also in the simulation results the residual tension in the inhomogeneous sample increases with decreasing sample volume. At the same time, the cavity size decreases and jumps to zero almost discontinuously at a volume such that the homogeneous system has a tension θ = 670 bar. Starting from this point, reducing the volume of the now homogeneous sample increases the sample pressure, following the *P*(θ) equation of state discussed in section A for the homogeneous sample. An important aspect to remark is that, despite the short relaxation time of 200 ps per point, the ⟨*N*⟩, (δ*V*), and θ(δ*V*) curves in Figure 7 are reversible, and each point represents an equilibrium value.

We emphasize again that the results apparently reflect the essential size dependence of pressure and surface free energy in the simulated samples. In an extended system, the diverging elastic energy would always overcome the surface tension contribution, and upon formation of the bubble, the residual tension would drop to zero.

### C Cavitation in IL/Water Solution

The investigation of cavitation reported in the previous subsection for pure water samples has been repeated in samples consisting of [Tea][Ms]/water and [P_4444_][DMBS]/water solutions, both at ∼25 wt % IL concentration. Samples at the critical θ_start_ = θ_crit_ and two subcritical θ_start_ < θ_crit_ tensions have been considered for each IL, spanning 100 bar. To compare properties of different systems, we carried out our analysis on systems with the same difference θ_crit_ – θ_start_ = 100 bar, hence, θ_start_ = θ_crit_ – 100 bar. The main parameters characterizing cavitation are reported in Table 2. As already stated, the stability under tension increases upon addition of [Tea][Ms] and decreases with [P_4444_][DMBS]. On the other hand, both with [Tea][Ms] and [P_4444_][DMBS], the duration of the cavity formation stage following nucleation increases with respect to the pure water case. The change is by a factor of 3 in the [Tea][Ms]/water case (see Figure S6 in the SI), and about eight in the [P_4444_][DMBS]/water case (see Figure S7 in the SI). The common feature of the two ILs that is likely to explain the similar effect in the two solutions is the increase in viscosity due to either ILs.

**Figure 8:**
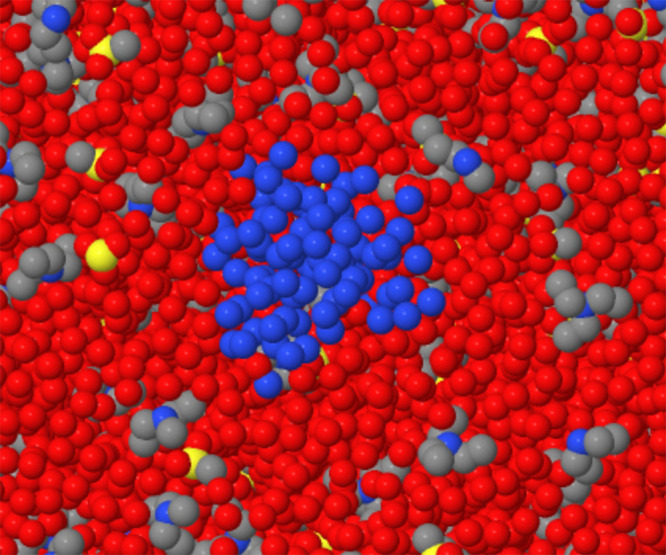
First nucleus of a cavity in [Tea][Ms]/water samples. Blue dots: oxygen belonging to water added to identify the cavity. Red dots: oxygen atoms in the solvation water. Black and yellow dots: non-H atoms belonging to [tea][Ms] ions.

**Figure 9:**
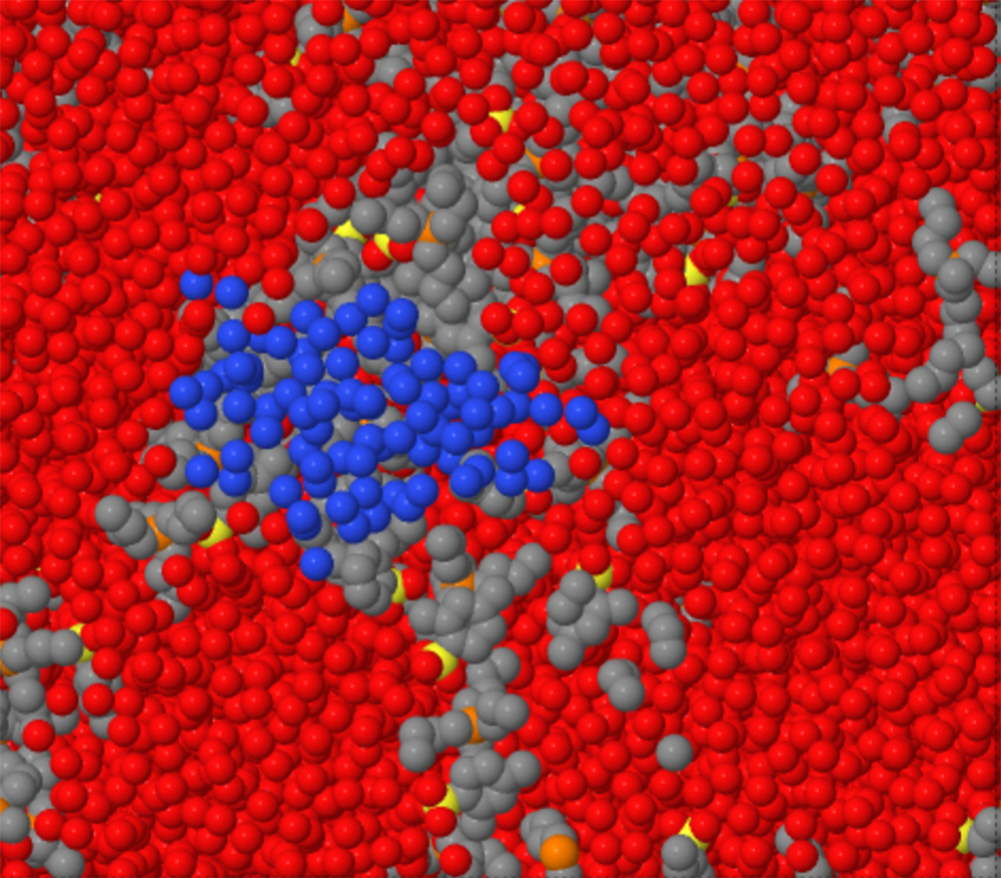
First nucleus of a cavity in [P_4444_][DMBS]/water samples. Blue dots: water added to identify the cavity. Red dots: oxygen atoms in the solvation water. Black and yellow dots: non-H atoms belonging to [P_4444_][DMBS] ions.

As already mentioned, the opposite effect on stability of the homogeneous state with respect to cavitation is likely to be due to the different solubility and surface activity of the two salts. For instance, [Tea][Ms] is rather soluble and does not behave like a surfactant. Then, the presence of the IL does not favor the formation of a solution/vapor interface. As a consequence, the cavity tends to form in contact with water, as shown in Figure 8. [P_4444_][DMBS] is less soluble, and at the temperature of the simulation and *P* = 1 bar, the [P_4444_][DMBS]/water solution is apparently nanostructured^64^ (see Figure S8 in the SI). Simulation results show that almost without exceptions the cavity forms at the interface between IL-rich and water-rich nanodomains, as shown in Figure 9. In some way, the cavity formation might be seen as heterogeneous nucleation, although the solution is nominally homogeneous, and the interfaces between the water-rich and the IL-rich domains are mesoscopic and not macroscopic.

As could be expected, the residual pressure is lower (higher tension) in the [Tea][Ms]/water case than for pure water, since both the surface tension at the solution/water vapor interface as well as the bulk modulus of the sample increase upon addition of the salt. The trend is less predictable for [P_4444_][DMBS]/water, since in this case the bulk modulus *B* increases and the surface tension decreases upon adding the salt. The increase of the residual θ_res_ after cavitation suggests that the change of *B* prevails, but the size of the effect points also to some other mechanism, due, for instance, to the fact that the solution/vapor interface is complex, being thick, highly structured, and has a strong cohesion because it contains both cations and anions with little water in between them. The complexity and stability of the interface are probably the reason why the stationary cavity in [P_4444_][DMBS]/water accounts for a smaller fraction of the excess volume (*V* – *V*_0_) than in water of [Tea][Ms]/water solutions.

**Figure 10:**
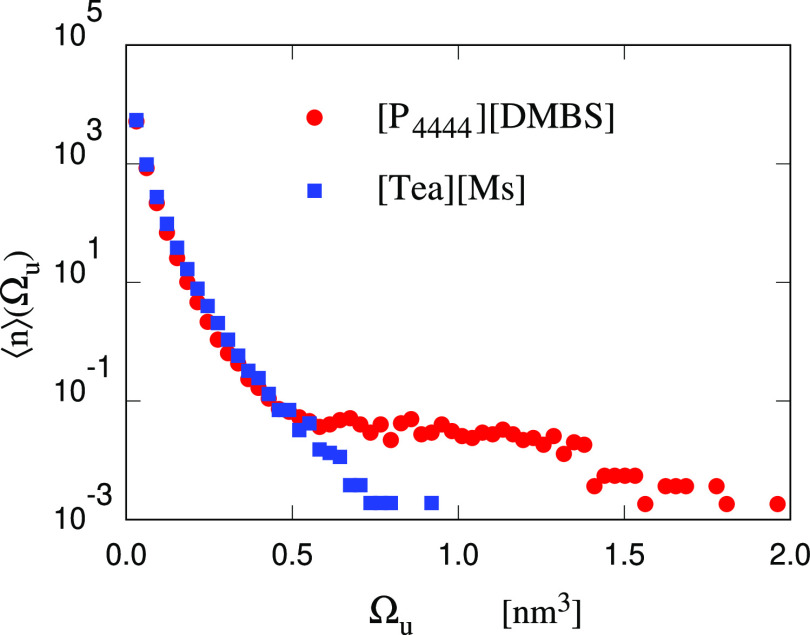
Average number of unstable cavities in the [P_4444_][DMBS]/water and [tea][Ms]/water samples as a function of cavity volume Ω_*u*_. The time average is carried out on the homogeneous samples before the nucleation of a stable cavity, considering 500 configurations separated by 2 ps from each other. In both samples the tension is 100 bar less than their respective θ_crit_.

The statistics of fleeting cavities in the homogeneous solutions prior to cavitation reflects these properties in an apparent way. The results shown in Figure 10 have been obtained on the two IL/water samples, both at θ = θ_crit_ – 100 bar. The results for the Small Water sample, not shown for reasons of clarity, are virtually intermediate between the two. The comparison shows that up to Ω = 0.5 nm^3^ the density of cavities as a function of size is nearly the same in the three samples. At larger Ω, however, the difference is significant, despite the difficulty in acquiring sufficient statistics for medium–large subcritical cavities. The distribution for [Tea][Ms]/water covers a much narrower range than in the other two cases. The distribution for [P_4444_][DMBS]/water shows a long tail extending almost flat to Ω = 2 nm^3^. The distribution for the Small Water sample, which is virtually indistinguishable from the one of the Large Water sample in Figure 10, is intermediate between the two IL/water cases. Although the difference in the three cases might not seem large, it is important to consider than the number of cavities is reported on a logarithmic scale. We verified that, in most cases, the bubbles of Ω > 1 nm^3^ in the [P_4444_][DMBS]/water sample form in contact with one of the IL-rich domains, and in some cases the IL-rich domain and the bubble are intimately compenetrated (see Figure S9 in the SI), confirming that the nucleation of the critical bubble is likely to occur at (mesoscopically) inhomogeneous conditions.

Analysis of snapshots and the computation of the inertia tensor show that the shape of the cavity in [P_4444_][DMBS]/water is significantly more irregular than in either pure water and [Tea][Ms]/water samples. Moreover, again in [P_4444_][DMBS]/water, fluctuations in size and shape of the cavity are more important than in the other two cases, reflecting the lower free energy of the solution/cavity interface.

Progressively reducing the sample volume in steps and equilibrating at constant volume in between the steps reveals different behaviors in the [Tea][Ms] and [P_4444_][DMBS] water solutions. The results for [Tea][Ms]/water, in particular, are similar to those obtained with water samples simulated according to the same protocol. The analysis starts from the sample with a stationary bubble at the volume *V* = 2418 nm^3^ of the homogeneous sample at θ = 1800 bar, reducing *V* in steps of 0.3%, relaxing each volume for 200 ps. By changing this duration, we verified that the 200 ps equilibration is already sufficient to provide time-independent values for the pressure and the cavity size. No specific segregation of organic ions at the cavity surface is observed in snapshots. At first, the size of the cavity decreases almost linearly with decreasing volume of the sample, while the sample pressure decreases faster than linearly. At the still sizable tension of θ̅ ∼ 900 bar, the cavity suddenly disappears (see Figure S10 in the SI). At lower tension, such that the system is homogeneous, the θ(*V*) relation is the same as measured in section A and shown in Figure 1, confirming the near equilibrium character of the points on the figure.

**Figure 11:**
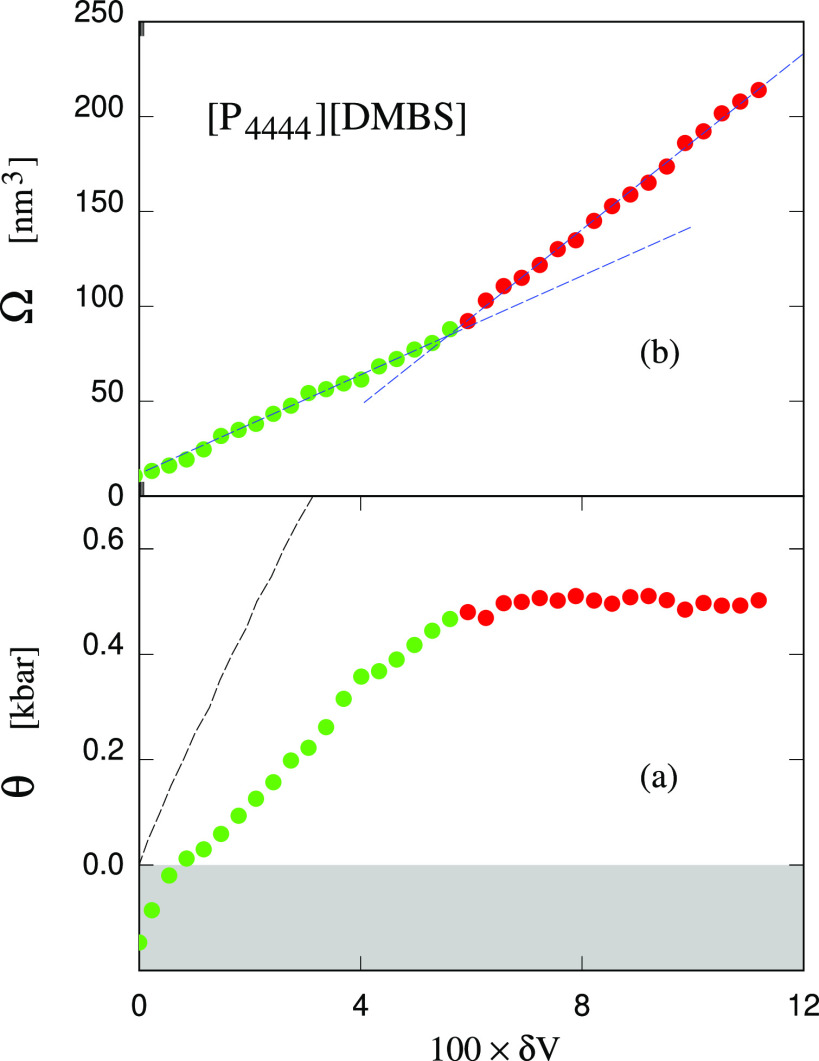
Tension in the sample and volume Ω of the stable cavity while squeezing the [P_4444_][DMBS]/water sample with a bubble inside, starting from the volume *V*_start_ = 2766 nm^3^ such that the homogeneous systems has a tension θ_start_ = 1250 bar. δ*V* = (*V* – *V*_0_)/*V*_0_, where *V*_0_ = 2488 nm^3^ is the equilibrium volume at *P* = 1 bar. Each dot represents a 400 ps NVT simulation carried out in sequence, moving from the right to the left of the figure. The green dots correspond to data greatly affected by the short (400 ps) equilibration time per point, thus deviating significantly from a reversible path. The straight dashed black line in panel (a) is the elastic θ(*V*) relation in the stretched homogeneous sample. The dashed blue lines in panel (b) highlight the broken linear behavior of the system properties as a function of excess volume. The light gray area corresponds to the overpressure required to squeeze out a bubble from the sample within ns time scales.

Applying the same volume reduction protocol, the [P_4444_][DMBS]/water sample behaves in a different way, as shown in Figure 11. In this case, the analysis starts at *V* = 2766 nm^3^, with a cavity of Ω = 214.7 nm^3^ and a residual tension θ_res_ = 499 bar. A preliminary remark is that, in this case, the relaxation of the cavity following the volume change becomes increasingly slow with decreasing volume of the sample and of the cavity. Part of the slowing down is due to the slow process of segregating ions at the interface, and of optimizing their structure. To compensate for this effect, the relaxation time between successive reductions of the volume has been increased to 400 ps, but we verified that below δ*V* = (*V* – *V*_0_)/*V*_0_ = 0.06, the data for the residual tension and the cavity volume cannot be satisfactorily equilibrated, even resorting to much longer times, at the limit of out MD capability. The data reported in Figure S11 of the SI, for instance, show that the less than 400 ps sufficient to relax the volume changes for δ*V* > 0.06 stretch to several ns already at δ*V* = 0.03. To emphasize this situation, the points in Figure 11 that depend on the squeezing rate are painted in green. These preliminary considerations highlight the major role of kinetics in determining the observations discusses for [P_4444_][DMBS]/water.

Then, the most important observation is that, with decreasing volume, the size of the cavity does not drop faster and faster, disappearing almost discontinuously at a tension θ̅, as seen in the previous two cases. Instead, the dependence of the cavity volume Ω on the excess sample volume (*V* – *V*_0_) changes continuously but rapidly when the data start to depend heavily on the relaxation time, and the full curve resembles the union of two linear segments joined at δ*V* = 6% (see Figure 11). This picture looks like the behavior of systems undergoing a glass transition, whose linear properties display a change of their slope as a function of temperature at the point at which the system leaves the equilibrium condition. This vitrification aspect will be briefly discussed again in relation to cavity collapse, based on the discrepancy between simulation data and the prediction of the RP equation.

**Figure 12:**
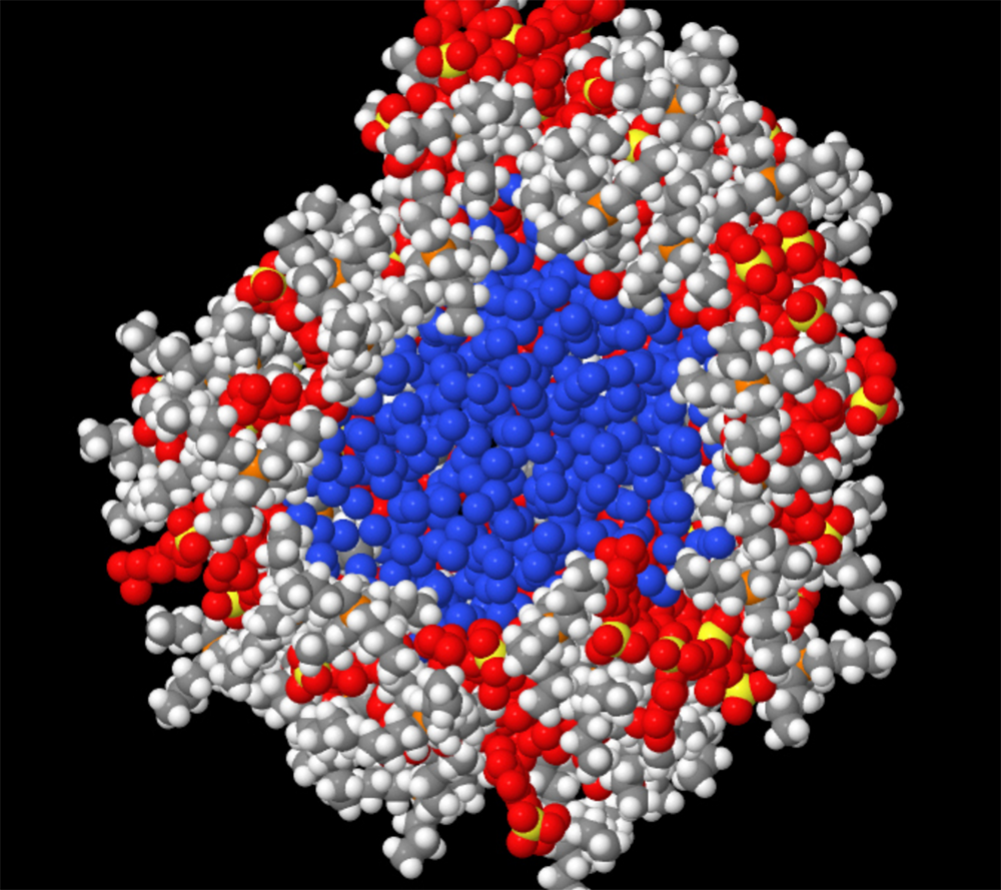
Onion-like structure of the bubble in [P_4444_][DMBS]/water solution collapsing upon shrinking volume. Normal colors for cation. Red: anions. Blue: void atoms. ions in the forefront have been removed to show the layering of components. Virtually no water within the radius considered in the picture.

Analysis of snapshots confirms that the slowing down to the point of vitrification concerns the organization of ions at the solution/water vapor interface. In a parallel way, the tension in the system does not show the initial increase with decreasing volume seen in the water and [Tea][DMBS]/water cases, and smoothly turns downward again at δ*V* ∼ 6%, remaining below the θ(*V*) equation of state of the homogeneous solution under tension. It turns out that a positive overpressure is required to bring the sample to the original volume before cavitation, as highlighted in Figure 11. We emphasize again that the reason for this anomalous behavior, contradicting the predictions of the simple model of eq 5 is the formation of a long-lived IL shell stabilizing the cavity, whose onion-like structure is shown in Figure 12. Two remarks are in order. First, the IL shell protecting the cavity is made of an equal number of cations and anions, with cations located closer to the vapor region than anions and very little water in the IL shell. Second, we point out that drastic slowing down of the kinetics concerns only the IL shell described in this paragraph. The rest of the system is liquid like, behaving like the nanostructured solution before stretching and cavitation, as confirmed by the computation of the water diffusion constant.

Together with the results of the following subsections, the data of the present one show that a suitable choice of the IL and some optimization of experimental conditions such as the frequency and the amplitude of the ultrasound wave can lead to relaxation times of the bubbles much longer than all other molecular processes in the same solution. In turn, this remark shows that, although the observations discussed in this section might be seen as minor details in the behavior of a complex system undergoing periodic cycles of expansion and compression, they also point to potentially useful applications,^65^ as discussed in the summary in section IV.

### D Bubble Collapse in Water

According to the RP eq 4, in an extended system at NPT conditions, a cavity will shrink and eventually dissolve at any pressure of the liquid environment *P*_out_ > −2γ (4π/3Ω)^(1/3)^, which, for bubbles of μm size, corresponds to a weak tension of the order of the bar. In our study, the collapse of bubbles has been simulated starting from a configuration with a bubble well equilibrated using NVT conditions, as described in the previous sections, at the sample volume and cavity size corresponding to θ_crit_. This choice of the initial box and cavity size has been made to compare the evolution of systems at corresponding states, that share the same property of being at the volume of θ_crit_ (for the homogeneous systems), and thus can be related to each other in some physically motivated way. Then, the collapse of the cavity has been triggered by reverting to NPT condition, with applied pressure (*P*_out_, in the notation of eq 4) ranging from zero to 300 bar. The change from NVT to NPT is necessarily sudden, possibly introducing some artifact in the time evolution. The analysis of pressure versus time in the sample, however, shows that the effect of the discontinuous change is limited to the first few ps of the collapse simulation, after which the evolution of all properties is smooth and stable (see Figure S12 in the SI).

In NPT simulations, the shrinking and collapse of the cavity are accompanied by a reduction of the box size that, in the samples simulated at the conditions described above, reaches up to 10% of the initial sample volume. Current approaches to enforce NPT condition aim at estimating correctly and efficiently thermodynamic properties, while the dynamics of the volume is admittedly ad-hoc. Popular methods such as the Parrinello–Rahman^66^ or Berendsen^67^ barostats introduce a time scale for the relaxation of pressure fluctuations which is largely arbitrary and usually is selected empirically to optimize the convergence of computed properties to their equilibrium value. Our simulation of bubble collapse, however, aims at describing a genuine time-dependent process, and the volume relaxation dynamics has to reflect the effect of the many-particle dynamics and the distribution of microscopic strain around the bubble. To this aim, we use the Parrinello–Rahman barostat with a target relaxation time τ_r_ selected in order to reproduce the time evolution of the volume as predicted by the RP equation in the *P*_out_ = 0 case, which is the state most sensitively dependent on the viscosity and surface tension of the system. The result of such a fitting of τ_r_ for the Small Water sample is reported in Figure S13 of the SI. The RP curve has been obtained using the experimental values of γ and η, and integrating the second order differential equation (eq 4) using the velocity Verlet algorithm. The agreement between the RP and simulation data is remarkable during most of the process, only the tail of the collapse being poorly reproduced. This last drawback is expected since the continuum approximation underlying RP cannot be valid at low cavity volumes. For the Small Water sample, the best fit (measured by a least-squares norm) is achieved with a relaxation time τ_r_ = 13 ps for the Parrinello–Rahman barostat, which is about 1 order of magnitude longer than the τ_r_ used in most NPT simulations. We empirically verified that the same τ_r_ can be used for the same sample at different pressure, while its value needs to be revised for different sample sizes and for systems such as the IL/water samples having a different viscosity and/or surface tension. For the Large Water sample, for instance, the optimal τ_r_ turns out to be 18 ps, as shown in Figure S14 of the SI.

**Figure 13:**
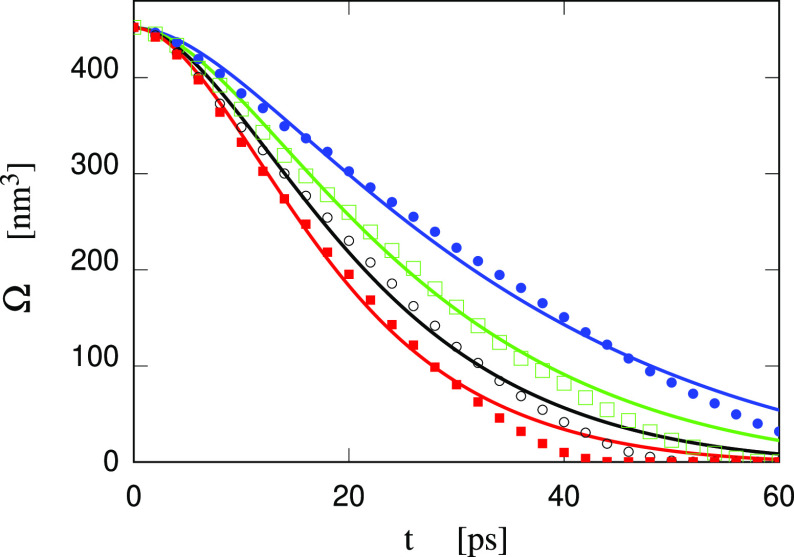
Volume Ω of the collapsing cavity under different pressures *P*_out_ of the liquid environment in the Small Water sample. Full lines are the results of the RP equation; symbols represent simulation data obtained at NPT conditions. All cases started from the same input configuration, corresponding to a bubble equilibrated at NVT conditions in the volume of θ_crit_ = 1400 bar for the homogeneous system prior to the cavity formation. Blue line and dots: *P* = 1 bar; green line and empty squares: *P* = 100 bar; black line and empty circles: *P* = 200 bar; red line and filled squares: *P* = 300 bar.

The ability of a single choice to cover a wide range of pressures on the liquid sample is illustrated in Figure 13. The good agreement based on a single fitting parameter of course represents also a validation of the RP theory down to nanometric size scales, to which simulation adds molecular details on the density and kinetic energy distributions, as well as information on the shape of the cavity and its fluctuations. The good agreement also implies that water follows hydrodynamic-type relations on the continuum down to very short scales, as already verified many times in the literature.

It might be useful to note that simulation provides a better and more reliable view of the final stages of the collapse, allowing also to test the sphericity of the collapsing bubble, and able to treat spherical and aspherical cavities on the same footing.

Analysis of bubbles collapsing in water, in particular, shows that their shape is virtually spherical at equilibrium, and becomes increasingly irregular with decreasing cavity size. In terms of duration and evolution of the cavity shape, therefore, the collapse stage appears to be the time-reversal image of the formation stage. Analysis of the kinetic energy of water molecules around the cavity shows a modest increase of local temperature (in the range of 10–20 °C), especially in the second half of the process. Because of the small cavity size, no statistics is available on the state of the vapor molecules inside the collapsing cavity, that is reality give origin to the plasma phase powering sonochemistry reactions and sonoluminescence. We emphasize again that this picture of the bubbles’ collapse, characterized by a smooth nearly monotonic convergence to equilibrium and moderate *Ṙ*(*t*) contraction rates, is certainly due to the small size of the simulated cavities, because RP predictions^2,49^ as well as the limited comparison of results for the Small and Large Water samples show that the collapse velocity, measured for instance by *Ṙ*(*t*), is proportional to the radius *R*(*t*) itself, provided the applied pressure during collapse is not vanishingly small.

### E Bubble Collapse in [Tea][Ma]/Water Solutions

The simulation of cavity collapse in [Tea][Ms]/water solutions has been carried out following the same protocol as for the water case. The sample already described in the previous sections has been turned discontinuously from NVT to NPT conditions, enforced through the Parrinello–Rahman barostat. Before switching from NVT to NPT, the ions appear uniformly distributed in the liquid fraction of the sample, and ions do not manifest any preference for the interface between the solution and the vapor bubble.

The calibration of the relaxation time τ_r_ of the barostat by comparison with the results of the RP equation requires an estimate of the viscosity and surface tension of the [Tea][Ms]/water solution, which are not available from experiments. To simplify the task, the relative change of viscosity has been inferred from the change of water diffusion in the two samples, relying on the Stokes–Einstein relation. The Stokes–Einstein relation by itself will not provide a quantitative value of η given the diffusion coefficient, but we expect that the relative variation of viscosity in going from water to [Tea][Ms]/water is more reliably predicted. The relative variation of surface tension has been estimated by comparing the amplitude of long-wavelength surface fluctuations again in water and [Tea][Ms]/water slabs, using the approach described, for instance, in ref (68). The computations reported in the SI show that the surface tension (Figure S15 in the SI) is increased by 7% and the viscosity increases (Figure S14 of the SI) by 72% upon adding [Tea][Ms] to water, at the relative concentration of 25 IL - 75 water wt %.

**Figure 14:**
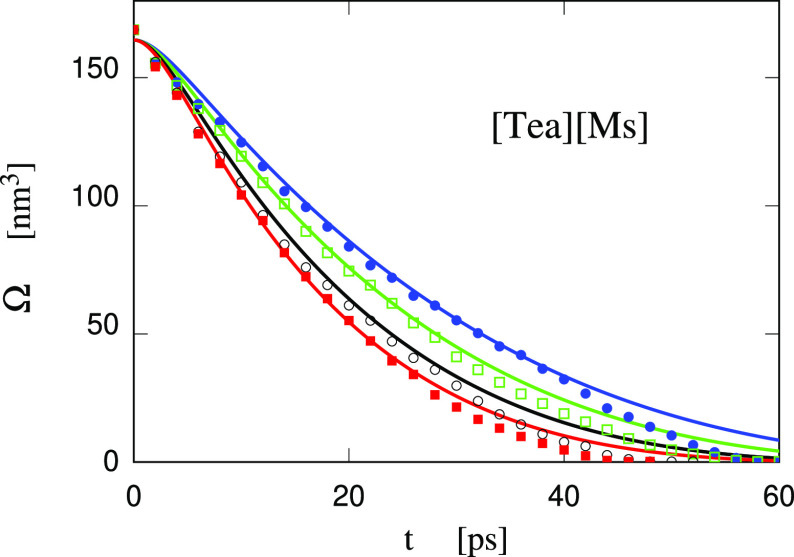
Comparison of simulation data with the results from the RP equation for the collapse of a bubble in [Tea][Ms]/water. The relaxation time of the simulation barostat has been set at τ_r_ = 13. All cases started from the same input configuration, corresponding to a bubble equilibrated at NVT conditions in the volume of θ_crit_ = 1800 bar for the homogeneous system prior to the cavity formation. Blue line and dots: *P* = 1 bar; green line and empty squares: *P* = 100 bar; black line and empty circles: *P* = 200 bar; red line and filled squares: *P* = 300 bar.

The results reported in Figure 14 show that the results from the RP equation and those from the simulation agree with each other at τ_r_ = 13 ps, equal to the value found for water. Apparently, the increase of γ (enhancing the shrinking) and of η (making the shrinking slower) compensate each other to some degree, and the overall size of the sample seems to be the determinant parameter. The agreement over most of the collapse confirms that the RP equation, based on the assumption of a homogeneous liquid phase, whose properties do not change upon cavitation or cavity collapse, provides a fair model of the process. A similar conclusion on the validity of the RP equation for simpler electrolyte solutions was reached in ref (63). Some disagreement between RP and MD-NPT, however, is apparent at very short times, because the Ω̇ = 0 boundary condition that we adopt (and that could be changed) does not correspond exactly to the starting point of the simulation, and in the tail of the collapse at *t* > 36 ps, where the continuum approximation is unable to correctly describe the molecular structure of the interface. Also, in this case, the formation and collapse stage appear to be specular copies of each other.

The effect of pressure is also similar to the one found for water, and the τ_r_ = 13 ps tuned on the *P* = 1 bar case is suitable for simulating the collapse also at higher pressure (see Figure 14).

### F Bubble Collapse in [P_4444_][DMBS]/Water Solutions

The simulation of cavity collapse in [P_4444_][DMBS]/water solutions has also been carried out following the same protocol as for the water case. As before, the sample has been turned discontinuously from NVT to NPT conditions, enforced through the Parrinello–Rahman barostat. At all stages, the nanostructuring of the sample is apparent already from simulation snapshots (see Figure S8 in the SI), and the surface of the equilibrated cavity is heavily decorated by ions, whose surfactant effect is apparent.

Also in this case, the calibration of the relaxation time τ_r_ of the barostat is attempted by comparison with the results of the RP equation. This, in turn, requires an estimate of the viscosity and surface tension of the [P_4444_][DMBS]/water solution which we carried out in the same way as for [Tea][Ms]. The computations reported in the SI show that the surface tension is decreased by roughly one-third and the viscosity increases by 80% upon adding [P_4444_][DMBS] to water, at the relative concentration of 25 IL wt % - 75 water wt %.

**Figure 15:**
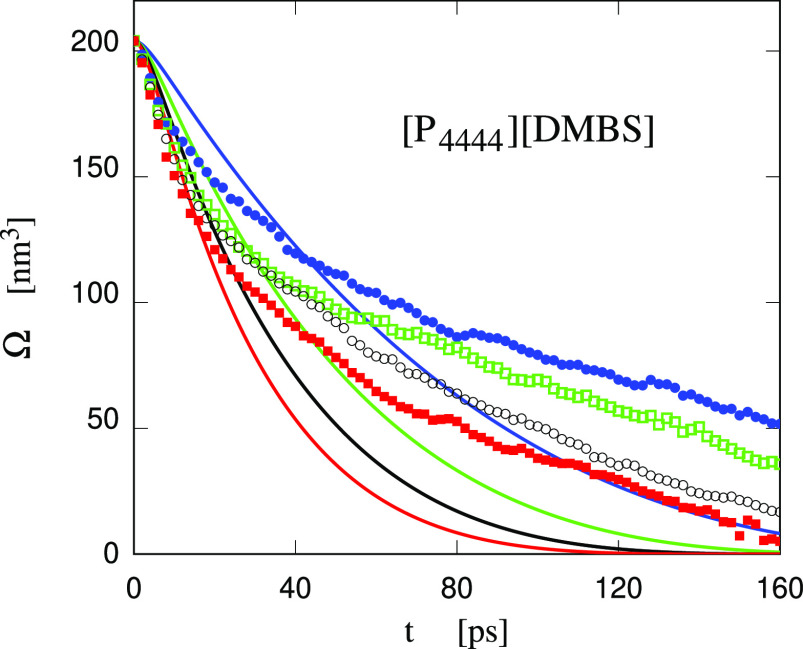
Volume Ω of the collapsing cavity under different applied pressures *P*_out_ on the [P_4444_][DMBS]/water sample. Full lines are the results of the RP equation; discrete symbols represent simulation data obtained at NPT conditions. All cases started from the same input configuration, corresponding to a bubble equilibrated at NVT conditions in the volume of θ_crit_ = 1250 bar for the homogeneous system prior to the cavity formation. Blue line and dots: *P* = 1 bar; green line and empty squares: *P* = 100 bar; black line and empty circles: *P* = 200 bar; red line and filled squares: *P* = 300 bar. The relaxation time of the simulation barostat has been set at τ_r_ = 13.

Using these data, the prediction of the RP equation for the cavity volume is qualitatively similar to the one for water, but stretches out to significantly longer times. Comparison of simulation data for a set of different τ_r_s, however, shows that the RP and simulation data cannot be brought into agreement. Figure 15, in which simulation data have been computed with the same τ_r_ = 13 ps used for water and [Tea][Ms]/water, in particular, shows that it is impossible to match the early and the medium-late stage at the same time, since the decay rate predicted by the RP equation is underestimated at short times and greatly overestimated at long times. Any change of τ_r_ will improve one side of the comparison and make the other worse. The qualitative deviation of collapse from the RP prediction reinforces the similarity with vitrification, since it points to a significant and systematic slowing down of relaxation with respect to a model that supposedly represents the paradigmatic evolution of a cavity in a simple fluid under a pressure imbalance.

The results of the previous sections, as well as the analysis of configurations, show that the reason for the disagreement is the formation of a long-lived surfactant shell at the interface between the cavity and the solution. In other terms, the RP equation cannot describe the volume evolution of bubbles in [P_4444_][DMBS]/water because the solution does not behave as a homogeneous medium whose properties do not depend on the nonequilibrium time evolution. The change of viscosity and surface tension in proximity of the cavity during the bubble collapse represents a deviation from a classical Newtonian fluid, that requires changes in the simple picture on which RP is based. The duration of the formation and collapsing stages is comparable, but the symmetry of the two stages is made ambiguous by the complex processes involved in the assembly and disassembly of the ionic layer at the cavity interface.

A strictly quantitative comparison of the collapse duration with the water case is not possible, since at the conditions of the simulation the cavity size in [P_4444_][DMBS]/water is one-half of that in water. Since at *P* = 0 the annihilation time is roughly proportional to size, the time imbalance between the two cases is even larger than what is shown in Figures 13 and 15.

Once again, the formation of long-lived mesoscopic structures in water provides the basis for possible applications in nanotechnology and drug delivery.

Application of pressure up to 300 bar speeds up the collapse of the cavity but does not change the general shape of the curve, which still presents long-time tails, nor does it improve the overall agreement with the RP equation (see Figure 15).

### G Competitive Evolution of Twin Bubbles

**Figure 16:**
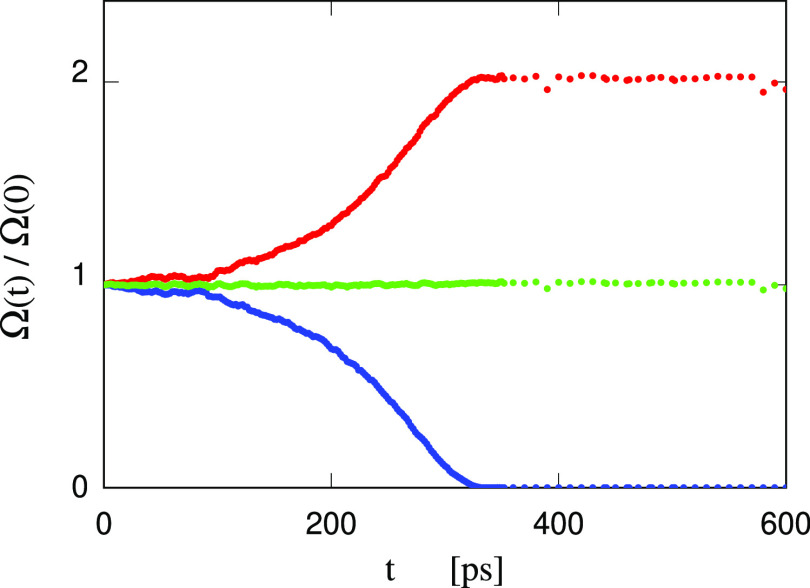
Time dependence of the volume of two cavities in water, initially of equal volume (see text). Red dots: growing cavity; blue dots: receding cavity; green dots: average of the two volumes. To ease comparison, all volumes have been scaled by the common volume of the two bubbles at the beginning of their evolution (*t* = 0).

The interaction of bubbles in a population has been described as competitive growth in ref (35), since the largest voids grow in time, at the expense of the smallest ones that disappear. To verify this picture and give some more quantitative detail, we took a sample with a stable nanometric void equilibrated for at least 10 ns, and we replicated it along the *x* direction displaced by a distance *L* equal to the periodicity of the original cubic system, obtaining an orthorhombic simulation sample of sides (2 × *L*; *L*; *L*) . Reassigning the particles’ velocity (extracting them from a Gaussian distribution at *T* = 300 K) gives two large voids of equal geometry and of equivalent but quantitatively distinct dynamics. In the case of water (Small Water sample), simulations started from the sample with a single stable bubble in the simulation volume of the homogeneous (metastable) water sample at θ = 1400 bar. Replication places the two bubbles of radius ∼4.5 nm at a center to center distance of 14.5 nm. Under their reciprocal influence, the bubbles do not drift toward or away from each other and do not coalesce by close contact fusion. Instead, after a short delay, the competitive growth mechanism sets in, with one of the two bubbles shrinking, and its volume is taken up almost exactly by the other one. As shown in Figure 16, the process is completed in about 300 ps, with only one stable bubble left in the system, of a volume twice as large as the initial one.

The duration of the noncontact coalescence is significantly longer than either the formation or the collapse of a bubble of the same size in water because in this process the total cavity volume (thus the elastic free energy of the liquid) is unchanged, and the driving force is only represented by the surface tension, favoring the largest bubble.

As expected, the time evolution of the twin bubbles in the [Tea][Ms]/water solution is quantitatively different but qualitatively similar to that of water, as shown in Figure S14 of the SI. In this case, the process is completed in about 400 ps, i.e., only slightly longer than in the water case, possibly due to the higher viscosity of the IL/water solution.

**Figure 17:**
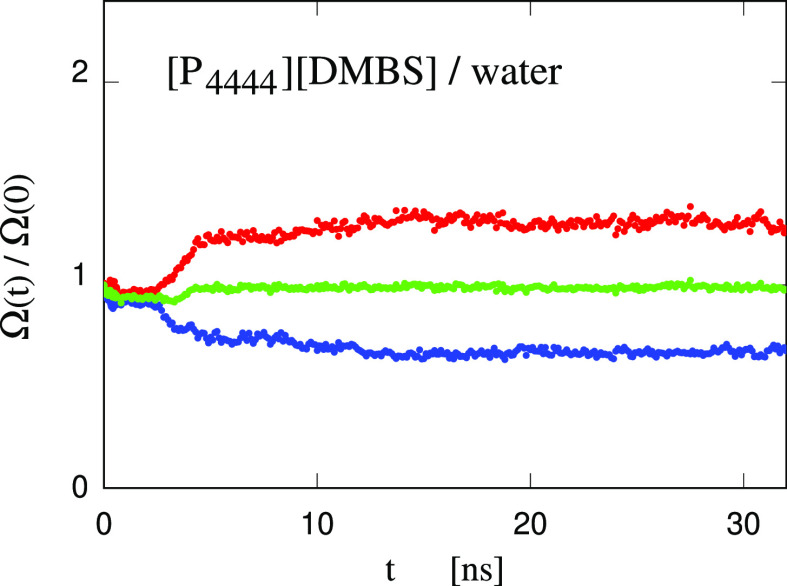
Time dependence of the volume of two cavities in the [P_4444_][DMBS]/water solution, initially of equal volume (see text). Red dots: growing cavity; blue dots: receding cavity; green dots: average of the two volumes. To ease comparison, all volumes have been scaled by the common volume of the two bubbles at the beginning of their evolution (*t* = 0). Moreover, the vertical scale [0:2] is the same of the pure water case in Figure 16. Note the change of scale for the time axis with respect to Figure 16.

Once again, the evolution of the twin bubbles is quantitatively but also qualitatively different in the [P_4444_][DMBS] case. First of all, the bubbles’ evolution is very slow since the beginning, partly because the lower surface tension provides a weaker advantage to the growing bubble. Moreover, when the smallest of the two bubbles is significantly smaller than the largest one, the population of ions at its surface becomes more tightly packed, slowing down even further the bubble evolution. Also, in this case, one observes a glassification of the ion shell that decorates the bubble interface. In practice, despite a 32 ns-long simulation, we have not been able to follow the process up to its termination. Judging from Figure 17, the incorporation of one cavity into the other by competitive growth will take at least 100 ns, but given the relatively narrow basis for the extrapolation, the estimate could also be greatly underestimated.

## IV Discussion and Conclusions

The nucleation and collapse of bubbles in a liquid due to the propagation of high-frequency ultrasonic waves underlie a variety of phenomena relevant to technology, chemical-physics, and medicine. Approaches able to affect and control these processes could contribute to new and better applications of ultrasonication. In the present study, the effect of two organic salts on the formation and collapse of bubbles in water driven by pressure waves has been investigated by atomistic molecular dynamics. MD in the NVT ensemble, in particular, has been used to investigate the nucleation, growth and equilibration of cavities in metastable homogeneous samples under tension. MD in the NPT ensemble, instead, has been used to determine the mechanical stability range of homogeneous samples under tension, and to investigate the collapse of bubbles when the pressure turns from negative to positive. Two organic-ionic compounds have been considered. The first one, i.e., [Tea][Ms] is relatively soluble in water, giving origin to solutions that are homogeneous down to the molecular scale. The second one, i.e., [P_4444_][DMBS] is more amphiphilic, and its water solutions are nanostructured, meaning that the overall homogeneous system consists of a complex arrangement of water-rich and IL-rich nanodomains. To provide a comparison for these two solutions, analogous simulations have been carried out also for water samples. The results of these tests for water show that, with minimal tuning, the Rayleigh-Plesset equation on the continuum is able to reproduce the NPT-MD results for the time dependence of the radius of spherical cavities in water, down to nanometric sizes. In particular, for the collapse of bubbles at *P* ≥ 0, the agreement of NPT simulation results with the predictions of the RP equation implies that for low applied pressure the time to annihilate a cavity is proportional to the cavity radius, while at moderate/high pressure the time is independent of the cavity size. The separation between the low and high pressure case is represented by the Laplace pressure 2γ/*R*, where *R* is the radius of the cavity. The NVT-MD results, instead, show some peculiar dependence on the size of the simulation sample not contained in the usual RP picture. These details, however, can still be understood and modeled using the same basic thermodynamic and continuum picture underlying the RP equation.

Further test computations for the two IL solutions and for pure water show that dissolving [Tea][Ms] in water at 25 wt % concentration increases the viscosity of the system by 70% and the surface tension by 7%. Dissolving [P_4444_][DMBS] in water at the same wt % concentration increases viscosity by 80% and decreases the surface tension by 33%. Because of these changes, the addition of the two ILs changes the stability with respect to cavitation and especially the kinetics of cavity formation and collapse. In particular, [Tea][Ms] increases the surface tension of water, making it more difficult to nucleate cavities, thus increasing the stability range of the homogeneous solution under tension with respect to water. The opposite is true with [P_4444_][DMBS], which acts as a surfactant and decreases the surface tension of water. In this case, the pressure stability range of the homogeneous solution under tension decreases, again with respect to water. In [P_4444_][DMBS]/water samples, the large, stable bubble that forms because of tension is invariably located at the surface of IL-rich domains. One could even say that the nucleation of cavities in [P_4444_][DMBS] solutions is heterogeneous since it always occurs at the interface between an IL and a water domain. In [Tea][Ms]/water solution, instead, bubbles preferentially nucleate in water, without a positive correlation with the location of ions. Then, apart from quantitative differences, the overall picture of bubble collapse in [Tea][Ms]/water solution follows the same RP picture observed for water. Once again, the [P_4444_][DMBS] case deviates significantly from these pictures. During the cavity collapse, the amphiphilic ions of [P_4444_][DMBS] become increasingly concentrated on the shrinking interface, creating a self-organized shell that resists to the collapse. While the collapse of nanometric bubbles takes the order of 50 ps in water, and a bubble of comparable size in [Tea][Ms] takes only slightly longer to collapse, it takes longer than 200 ps in [P_4444_][DMBS]/water solution. Even an applied pressure of 300 bar is not enough to curtail the long persistence of small cavities protected by an interfacial shell of [P_4444_][DMBS]. As is apparent from these observations, the time evolution of the bubble size during this process deviated qualitatively from the results of the RP equation. This is not surprising, since the homogeneity assumption underlying the RP equation is no longer satisfied, and the IL/water mixture acquires characters of a non-Newtonian fluid, since its surface tension, composition, and viscosity around the bubble change during its shrinking. Similar effects concern the interaction of bubbles of different sizes in [P_4444_][DMBS]/water solutions. While in water and in [Tea][Ms]/water solutions bubbles evolve on the 10^2^ ps scale according to the so-called competitive growth mechanism,^35^ in [P_4444_][DMBS]/water solutions, the equilibration of a population of bubbles is slowed down and perhaps prevented by the formation of a compact, impermeable shell of ions at the water/cavity interface. This effect results in the persistence of medium-small cavities for long times, possibly encompassing successive compression–expansion cycles. The stability of bubbles can be enhanced by aging effects due to the thickening and better organization of the ionic shells with increasing time. Then, simulations (not reported here) show that, because of the spontaneous nanostructuring of [P_4444_][DMBS]/water systems, the dissolution of IL-rich domains has, at best, a negligible free energy driving force, and therefore it is very slow.

From the application point of view, long-lived cavities in the liquid phase present a number of appealing opportunities.^65^ Lifetimes on the ns, or even μ scale, might not be sufficient for many applications, but our results have been obtained without any optimization in the choice of the IL compound nor of the formation and collapse conditions. Considering larger initial bubbles, more suitable ILs, and perhaps adding a jellifying third component, might easily extend the lifetime of cavities into the multi-μs range that could be sufficient for novel applications. A lifetime of several μs would cover several oscillations of the sound wave, thus leading to a population of stationary voids in the liquid. Voids of this type, in turn, could store gases (CO_2_, O_2_) dissolved at oversaturation concentrations in the liquid, and could enhance the ability of fluids and nanofluids to remove CO_2_ or carry molecular oxygen in artificial blood preparations,^69,70^ but could also represent nonequilibrium complex fluids suitable for chemical processing and for food technology.

## Supporting Information Available

A series of figures on structural, thermodynamic and dynamic properties of bubbles’ formation and collapse in water and in water solutions of [P_4444_][DMBS] and [Tea][Ms]; two short sections concerning the application of the Rayligh-Plesset equation to the collapse of bubbles in simulated systems (PDF)
A sequence of snapshots in *xyz* format documenting the formation of a bubble in water under tension (XYZ)
A sequence of snapshots in *xyz* format documenting the failed nucleation event in a homogeneous water sample under high tension (XYZ)

## Supplementary Material

jp2c07950_si_001.pdf

jp2c07950_si_002.xyz

jp2c07950_si_003.xyz
