# Supplementary material for: Effect of Organic Ions on The Formation and Collapse of Nanometric Bubbles in Ionic Liquid/Water Solutions: A Molecular Dynamics Study
Source: J Phys Chem B. 2023 Feb 14;127(7):1628–44. doi: 10.1021/acs.jpcb.2c07950 (PMC9969518; doi:10.1021/acs.jpcb.2c07950)
Supplement: Supplementary file 1 — jp2c07950_si_001.pdf [file jp2c07950_si_001.pdf]

Supporting Information for Publication:

The Effect of Organic Ions on The Formation and Collapse of Nanometric Bubbles in Ionic Liquid/water Solutions: A Molecular Dynamics Study

Raffaela Cabriolu[(1)*], Bruno G. Pollet [(2)] and Pietro Ballone[(3,4)]

(1) Department of Physics, Norwegian University of Science and Technology (NTNU), 7491 Trondheim

(2) Green Hydrogen Laboratory, Université du Québec à Trois-Rivières, 3351 Boulevard des Forges, Trois-Rivières, QuebecG9A 5H7, Canada

(3) School of Physics, University College, Dublin, Ireland and

(4) Conway Institute for Biomolecular and Biomedical Research, University College, Dublin, Ireland

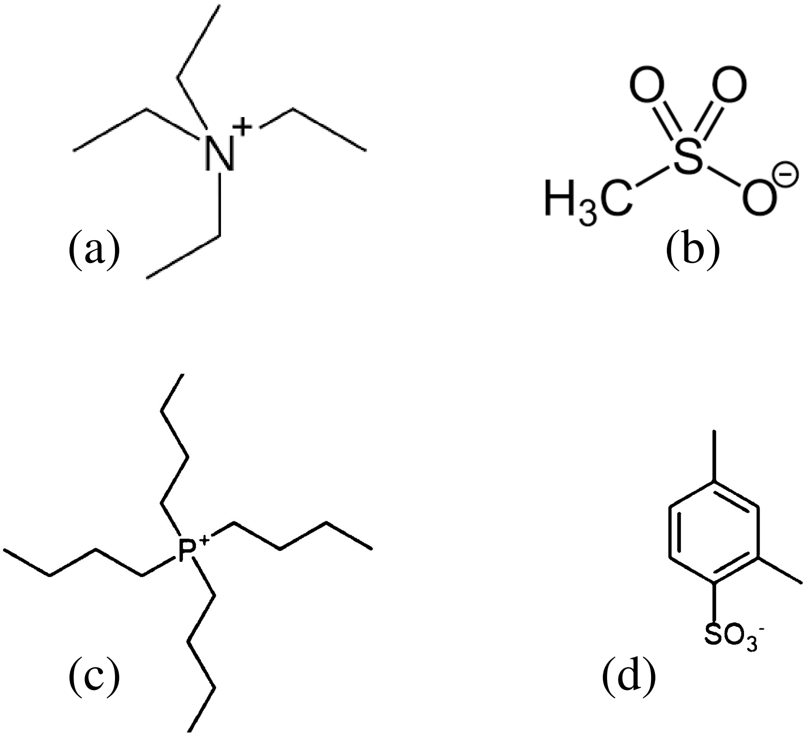

FIG. S1: Schematic structure of the ions considered in the present study. (a) $[Tea]^{+}$; (b) $[Ms]^{-}$; (c) $[P_{4444}]^{+}$; (d) $[DMBS]^{-}$.

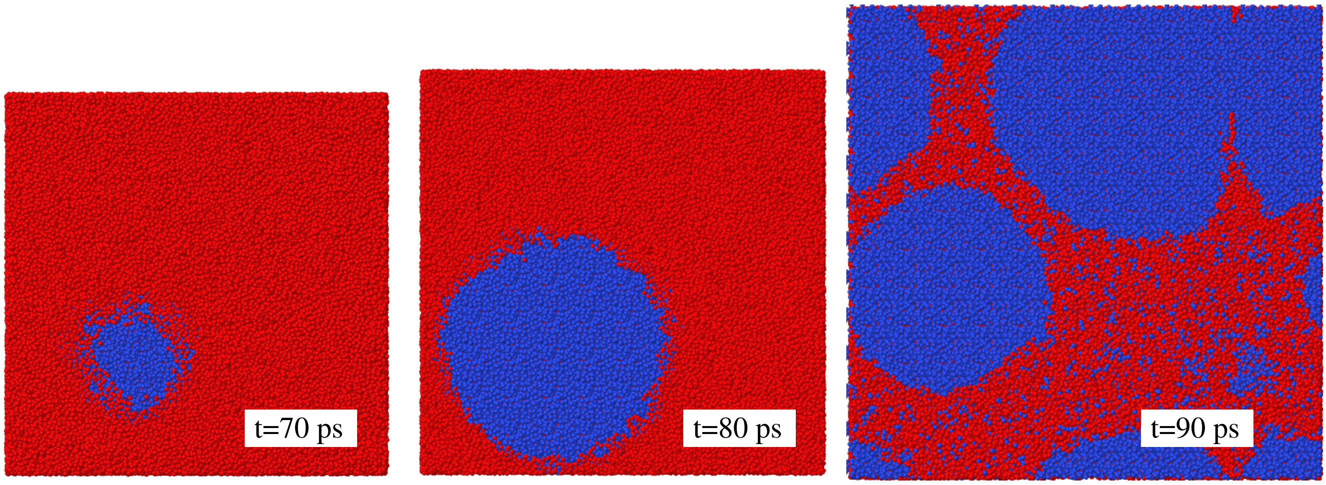

FIG. S2: Snapshots from the NPT simulation of a water sample consisting of 200×10^3 water molecules under a tension $\theta = -P = 1400$ bar. The sequence illustrates the sudden collapse of the sample following the nucleation of a bubble. At $t = 90$ ps the sample has lost all ability to resist the external tension, and its volume is expanding at a rapidly increasing rate. To prevent such an unbound expansion, the analysis of bubble nucleation, growth and bubble-bubble interaction have been investigated at NVT conditions. Red dots: OW atoms of water. Blue dots: oxygen of the ghost particles inserted to identify voids in the system (see text). Hydrogen atoms not shown. The side of the sample in the three panels is proportional to the size of the simulation cell at the times reported in the figure.

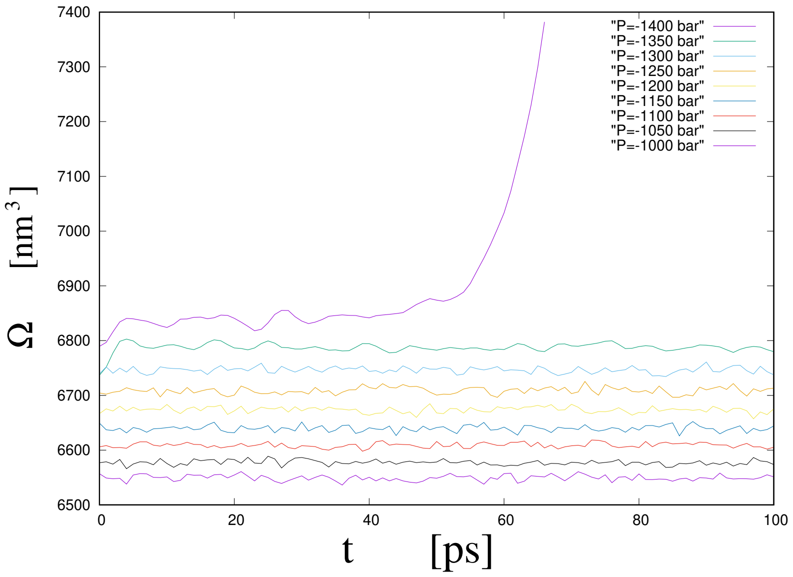

FIG. S3: Plots spanning the tension stability range of the large water sample. NPT simulations lasting 100 ps each. The curve with the largest volume variation identifies the critical tension with respect to cavitation $\theta_{crit} = 1400$ bar.

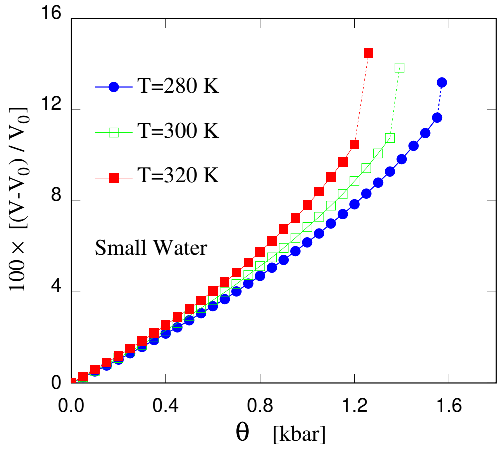

FIG. S4: $V(\theta)$ phase diagram comparing the stability range of pure water samples with respect to cavitation at $T = 280$ K, 300 K and 320 K.

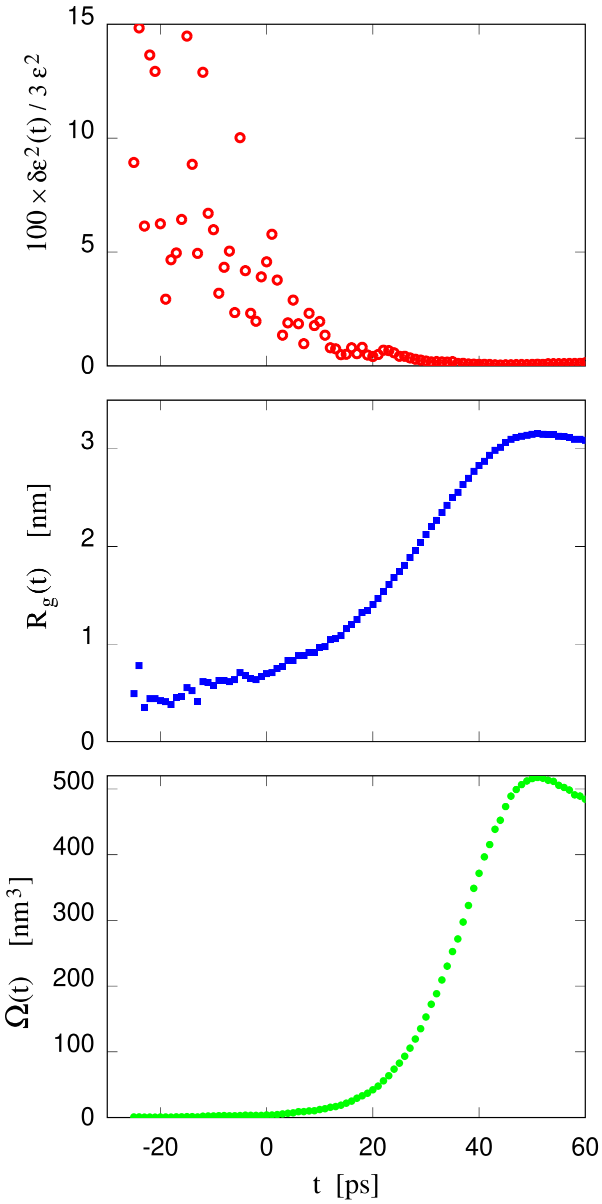

FIG. S5: Volume $\Omega(t)$ and shape parameters derived from the computation of the momenta of inertia of the cavities during formation of a bubble in the Large water sample. NVT simulations in the volume of the homogeneous sample at $\theta = 1300$ bar. The computation of the shape parameters is based on the N discrete volume elements V_{w0} corresponding to the N probe water molecules inserted in the sample to identify cavities in the system (see the Methods section in the main text). The gyration radius R_g is defined as $R_g(t) = \sqrt{\epsilon(t)/N(t)}$. The parameters ϵ and $\delta\epsilon^2/3\epsilon^2$ are defined in the text. $\delta\epsilon^2/3\epsilon^2$ in particular is a measure of the asphericity of the cavity.

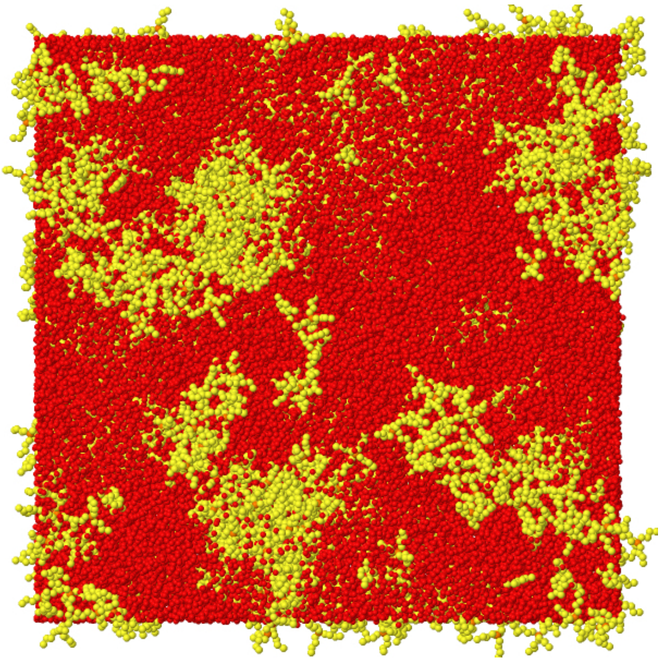

FIG. S8: Snapshot of the [P_{4444}][DMBS]/water sample at $T = 300$ K, $P = 1$ bar, and $25 - 75$ wt% IL-water composition, showing the marked nanostructuring of the sample, due to the formation of IL-rich and water-rich domains. Water domains are almost pure, while IL domains contain 10 wt% water. For illustration purposed, C atoms on the IL ions have been painted yellow, water O is red, H atoms are not shown.

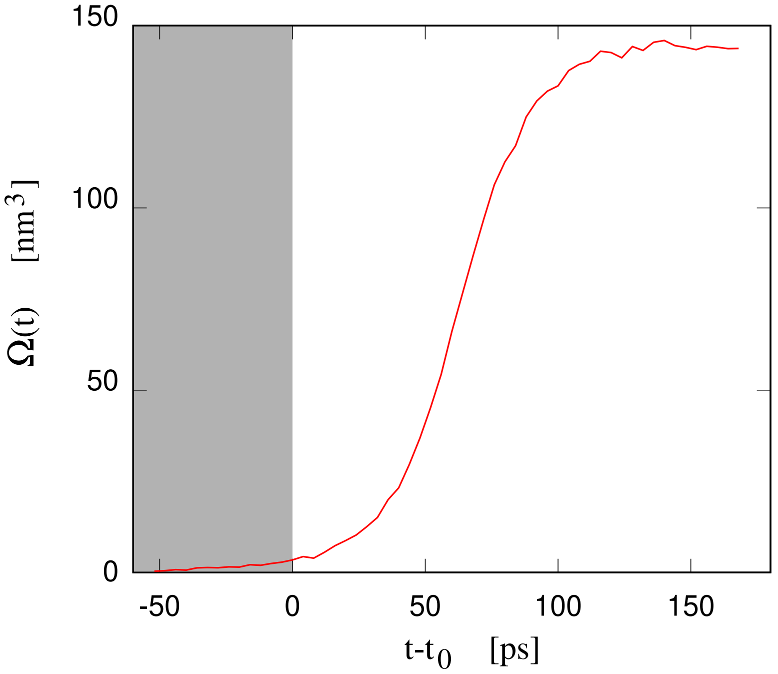

FIG. S6: Volume of the largest void in the [Tea][Ms]/water sample. NVT simulation at the sample volume $V = 2617$ nm^3 of the homogeneous system at $\theta = 1700$ bar, $T = 300$ K. The origin of the time scale corresponds to the τ_f at which the nucleation of the bubble manifests itself in an upward drift of the sample pressure. The analysis of cavities reported in this figure shows that nucleation started about 40 ps before, with the slow ripening of the initial nucleus (identified by the gray area at $t < 0$). Including this initial stage, the cavity formation lasts $\sim$ 180 ps, i.e., three times longer than in the water sample of the same size and equal $\theta_{crit} - \theta$.

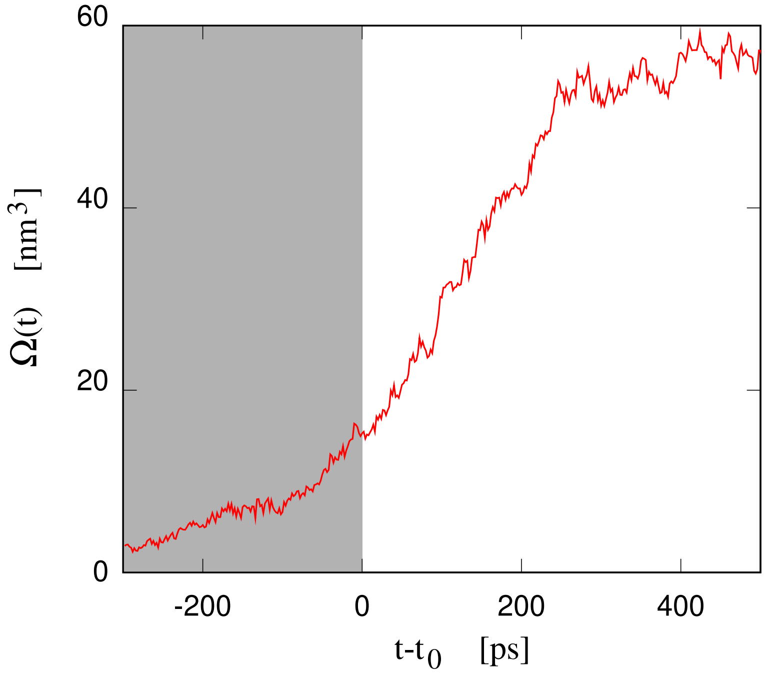

FIG. S7: Volume Ω of the largest void in the [P_{4444}][DMBS]/water sample. NVT simulation at the sample volume $V = 2626$ nm^3 of the homogeneous system at $\theta = 1150$ bar, $T = 300$ K. The origin of the time scale corresponds to the τ_f at which the nucleation of the bubble manifests itself in an upward drift of the sample pressure. The analysis of cavities reported in this figure shows that nucleation started at least 300 ps before, with the slow ripening of the initial nucleus (identified by the gray area at $t < 0$). Including this initial stage, the cavity formation lasts $\sim$ 500 ps, i.e., eight times longer than in the water sample of the same size and equal $\theta_{crit} - \theta$.

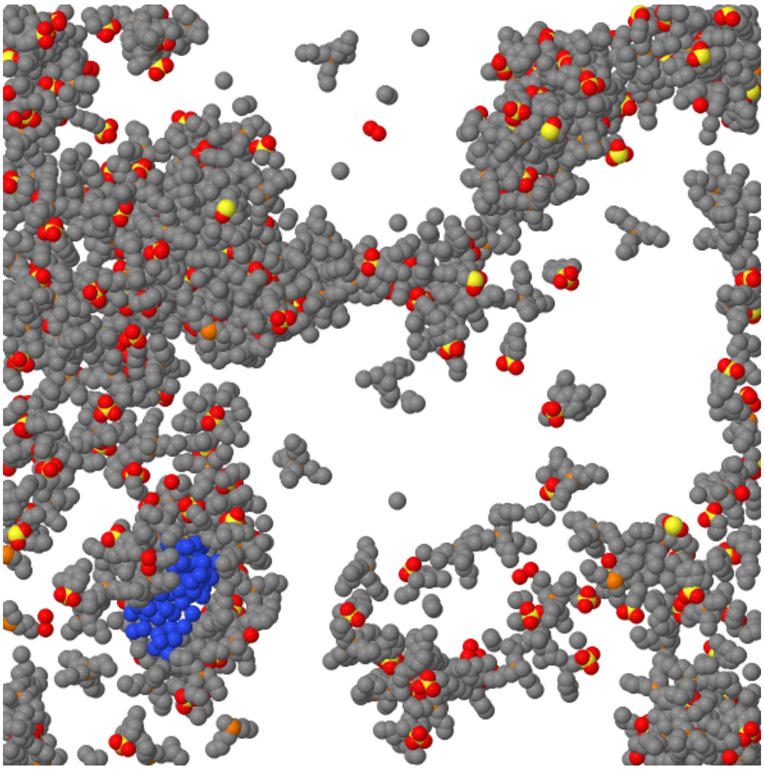

FIG. S9: Snapshot of a slice of the $[P_{4444}]$[DMBS] / water sample selected to illustrate the close relationship of nanostructuring and bubble formation. Black dots: carbon belonging to the ions; red dots: oxygen belonging to the ions; yellow dots: sulfur in the anions; orange dots: phosphorus in the cation. The blue dots represent the largest cavity in the sample, which is growing following nucleation.

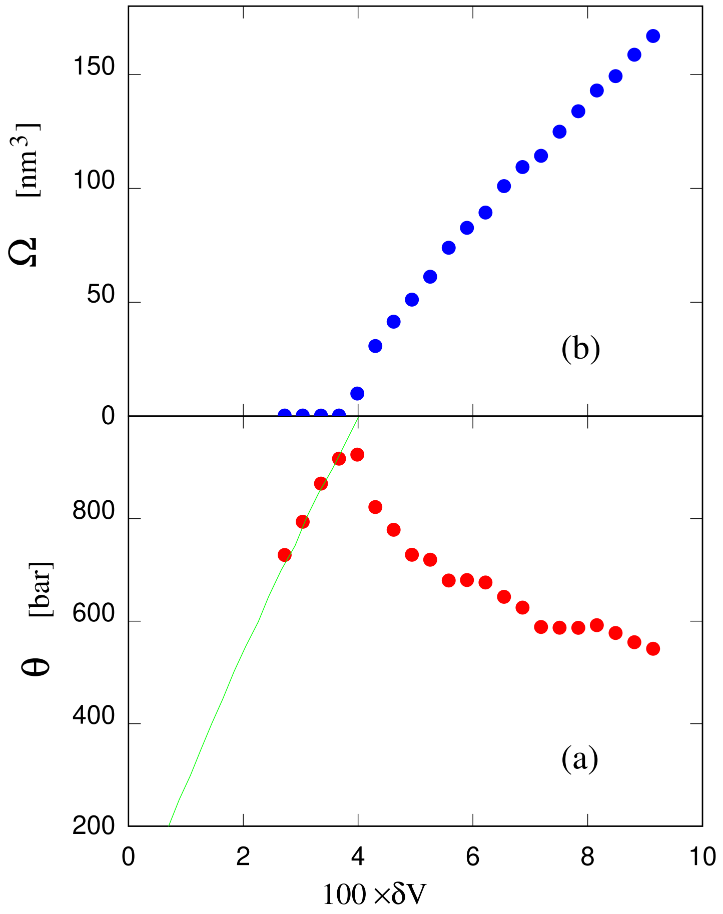

FIG. S10: [Tea][Ms]/water sample: Tension in the system and volume Ω of the largest cavity while squeezing the simulation box with a bubble inside, starting from the volume such that the homogeneous systems has a tension $\theta_{start} = 1800$ bar. $\delta V = (V - V_0)/V_0$ where $V_0 = 2418$ nm^3 is the equilibrium volume at $P = 1$ bar. Each dot represents a 200 ps NVT simulation carried out in sequence, moving from the right to the left of the figure. The straight dash line is the elastic $\theta(V)$ relation in the stretched homogeneous sample.

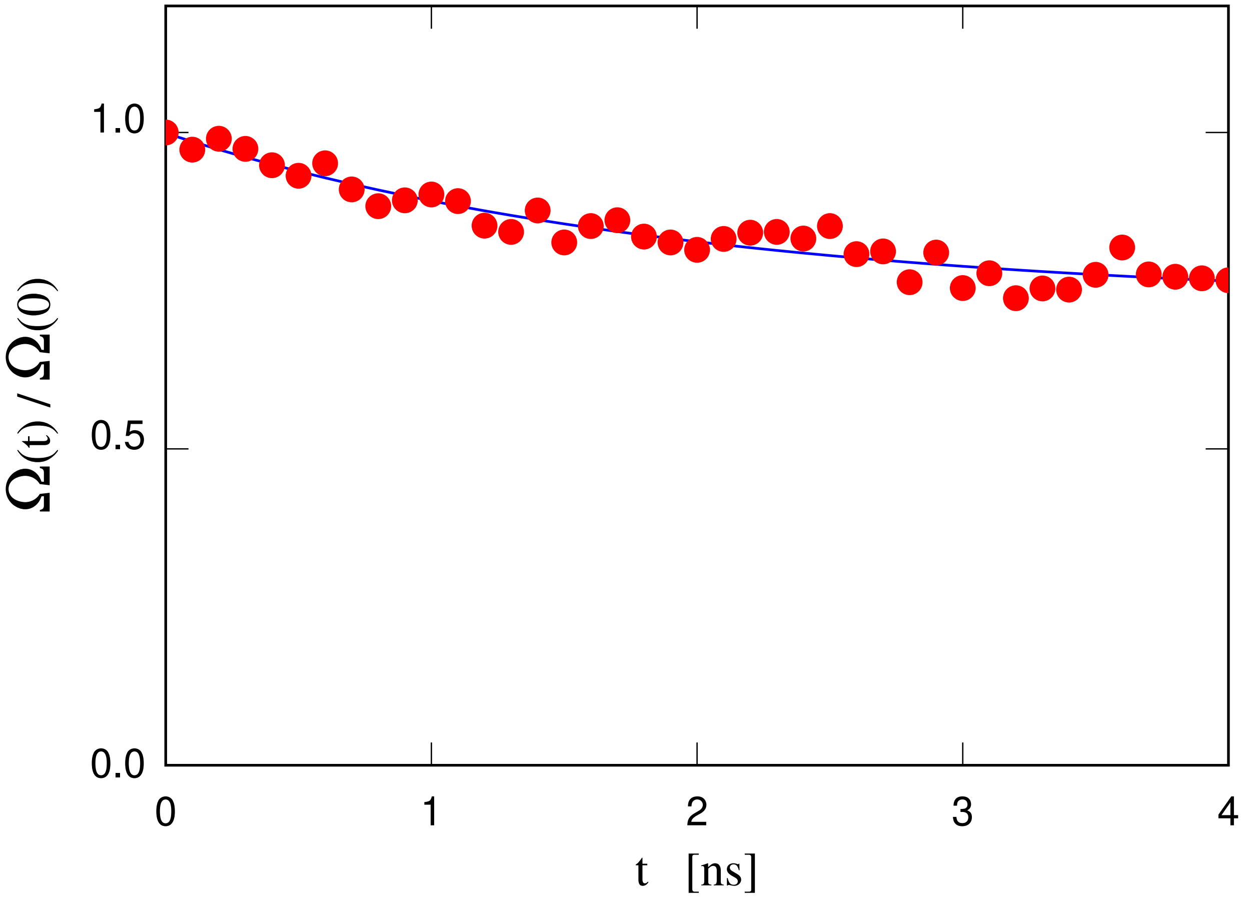

FIG. S11: Time dependence of the largest cavity in the $[P_{4444}]$[DMBS] sample following the discontinuous decrease of volume from $\delta V = 0.034$ to $\delta V = 0.031$, where $\delta V = (V - V_0)/V_0$ and $V_0 = 2766$ nm^3. Red dots: simulation data; blue line: fit of the simulation data by $V(t)/V(0) = A + B \exp(-t/\tau)$ with $\tau = 2$ ns. Full relaxation takes several τ.

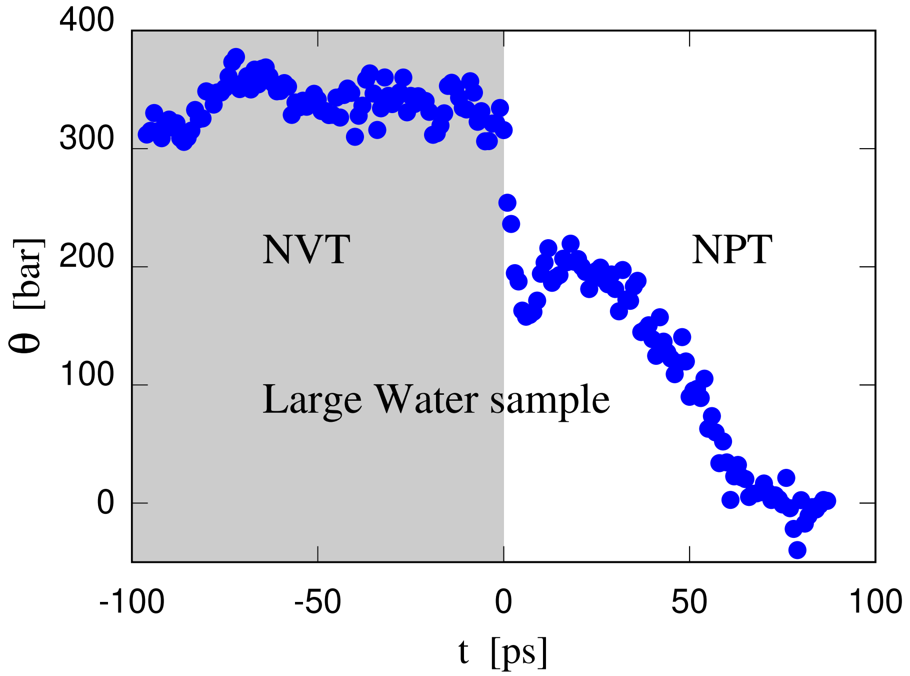

FIG. S12: Tension in the Large Water sample across the change from NVT and NPT conditions, taking place at $t = 0$, and during the collapse of the bubble at $t > 0$. At any $t > 0$, the system is subject to an external pressure $P = 1$ bar.

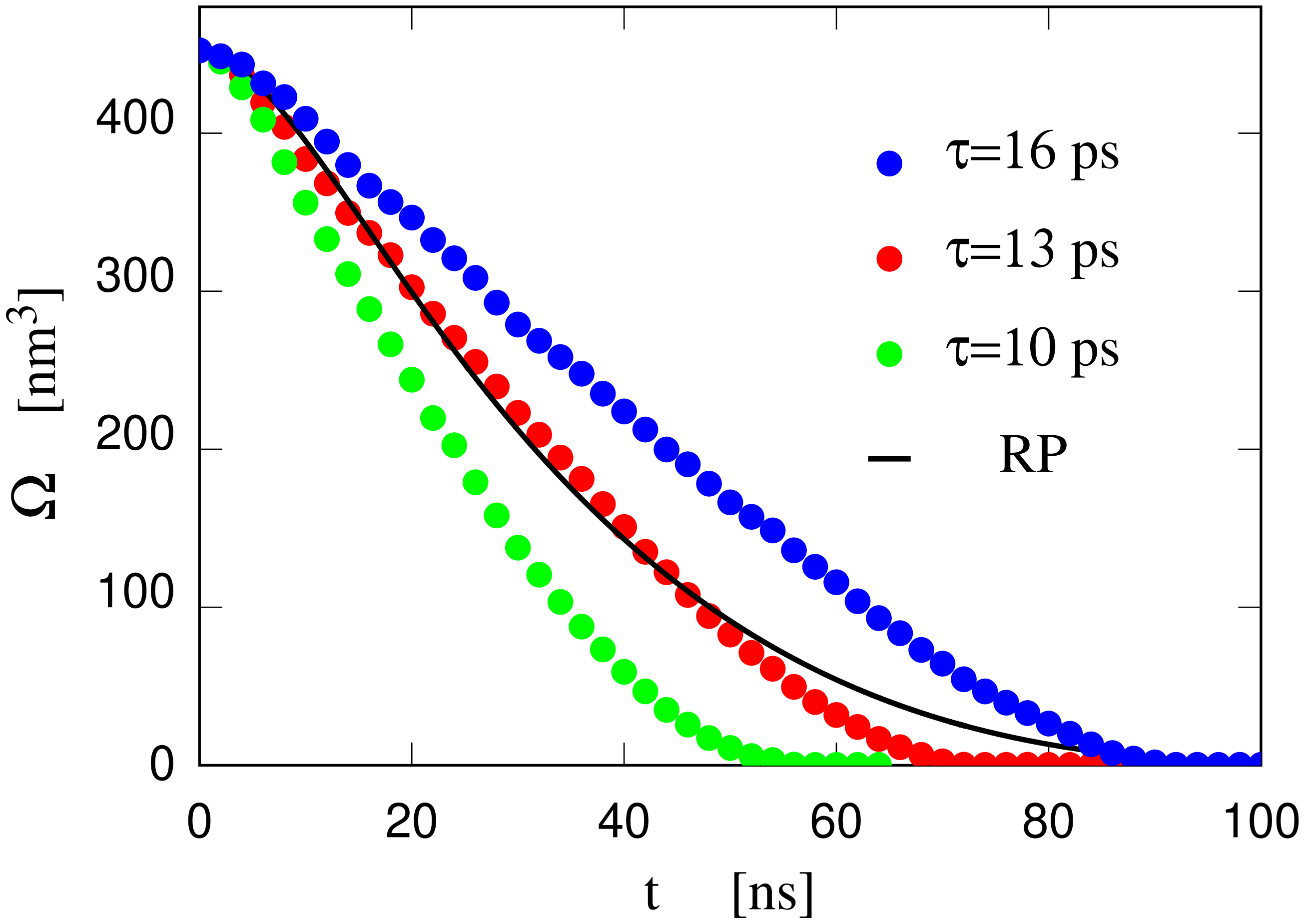

FIG. S13: Comparison of simulation data with the predictions of the RP equation for the collapse of a bubble in the Small Water sample. τ is the relaxation time constant in the Parrinello-Rahman barostat use in the NPT simulation. On the basis of these data, the relaxation time of the simulation barostat has been set to 13 ns.

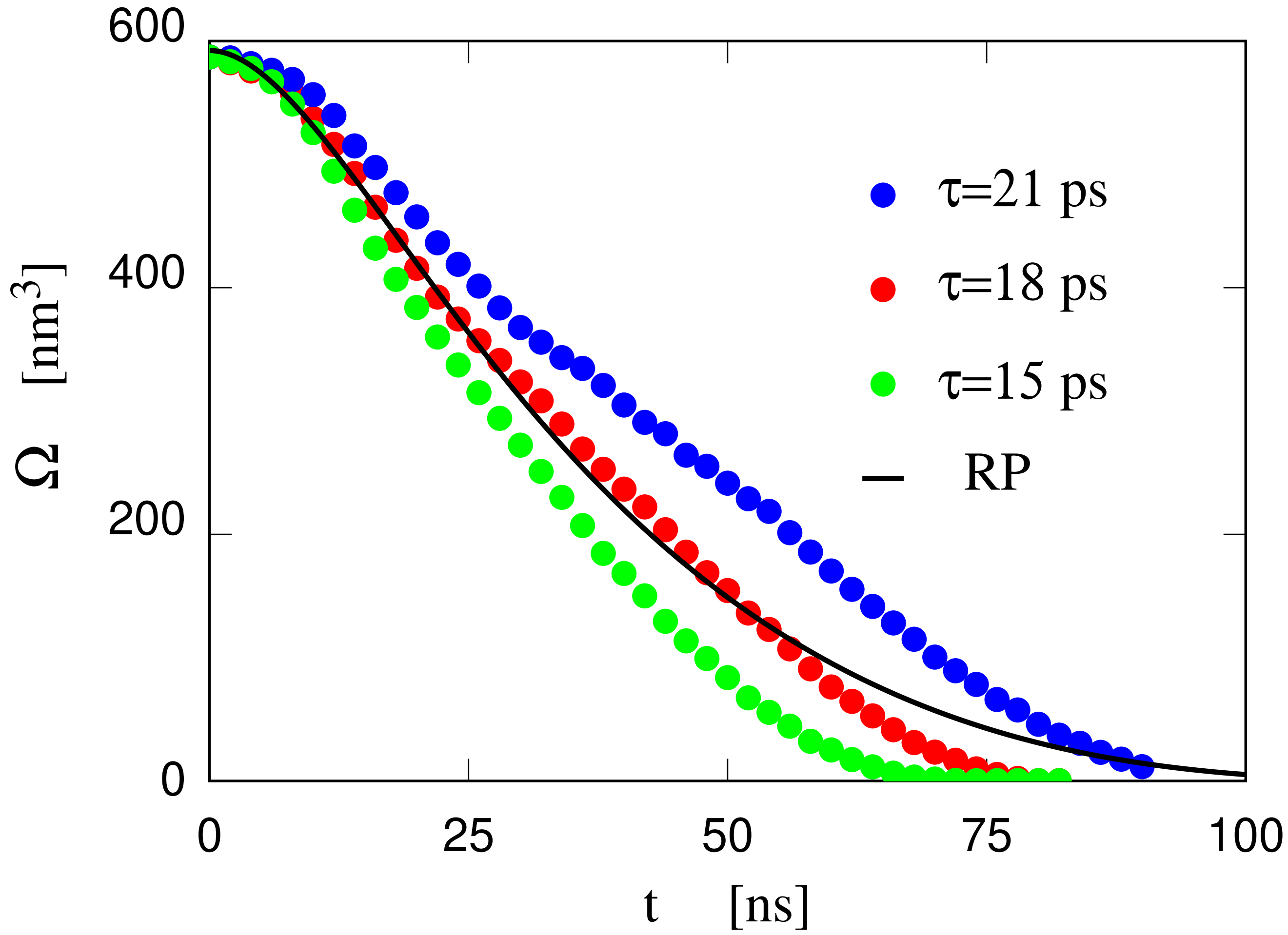

FIG. S14: Comparison of simulation data with the predictions of the RP equation for the collapse of a bubble in the Large Water sample. τ is the relaxation time constant in the Parrinello-Rahman barostat use in the NPT simulation. On the basis of these data, the relaxation time of the simulation barostat has been set to 18 ns.

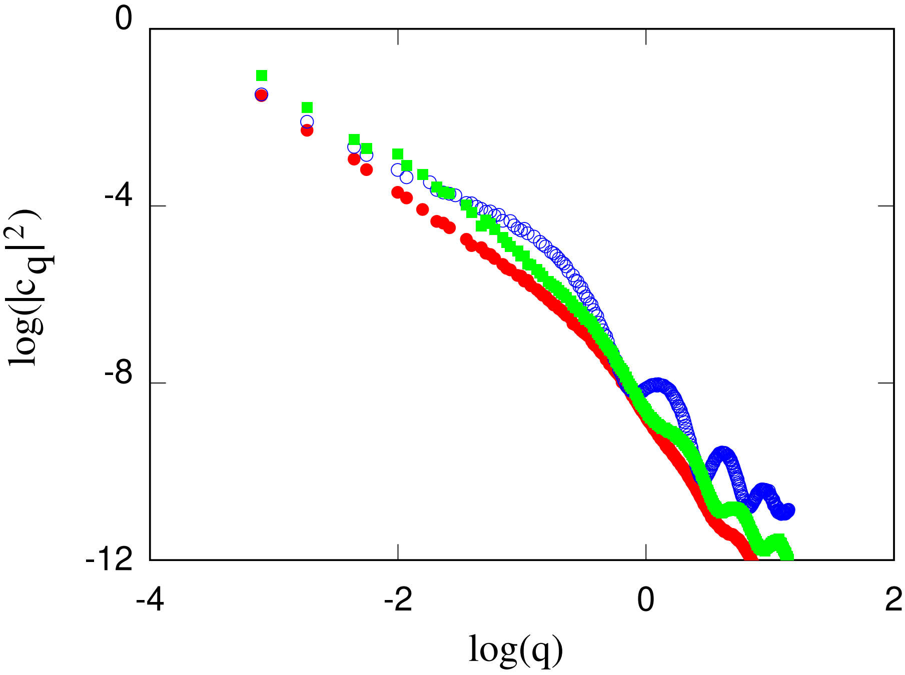

FIG. S15: Equilibrium fluctuations of the surface of water and IL solutions. The C_q quantity is computed as follows. First, the instantaneous surface $x(y, z)$ in the (y, z) plane is expressed as $x(y, z) = \sum_{\mathbf{q}\in\Omega} C_{\mathbf{q}} e^{i\mathbf{q}\mathbf{r}_{yz}}$ following the reference given in the text. Then, the result is averaged over hundreds of configurations to obtain $h(q) = \langle |C_q|^2 \rangle$. Fit of $h(q)$ with the function $h(q) = (k_B T/A_0)/(\gamma q^2 + k_c q^4)$ allows to estimate the surface tension γ and the bending rigidity K_c, as detailed, for instance, in Ref. 68 of the main text. In these expressions, $\mathbf{q}$ are 2D reciprocal lattice vectors of the simulation box along the (y, z) plane of the surface, A_0 is the average surface area, k_B is the Boltzmann constant, and T the sample temperature. Ideally, all curves of this type have the same slope -2 in the limit $q \to 0$. Deviations from this rule are due to the fact that presence of the vapour phase blurs the precise definition of the surface. Nevertheless, we estimate that the ratio of surface tension γ in water, [Tea][Ms] and [P_{4444}] is 1 : 1.07 : 0.66. Statistics is not sufficient to estimate a bending rigidity with acceptable accuracy.

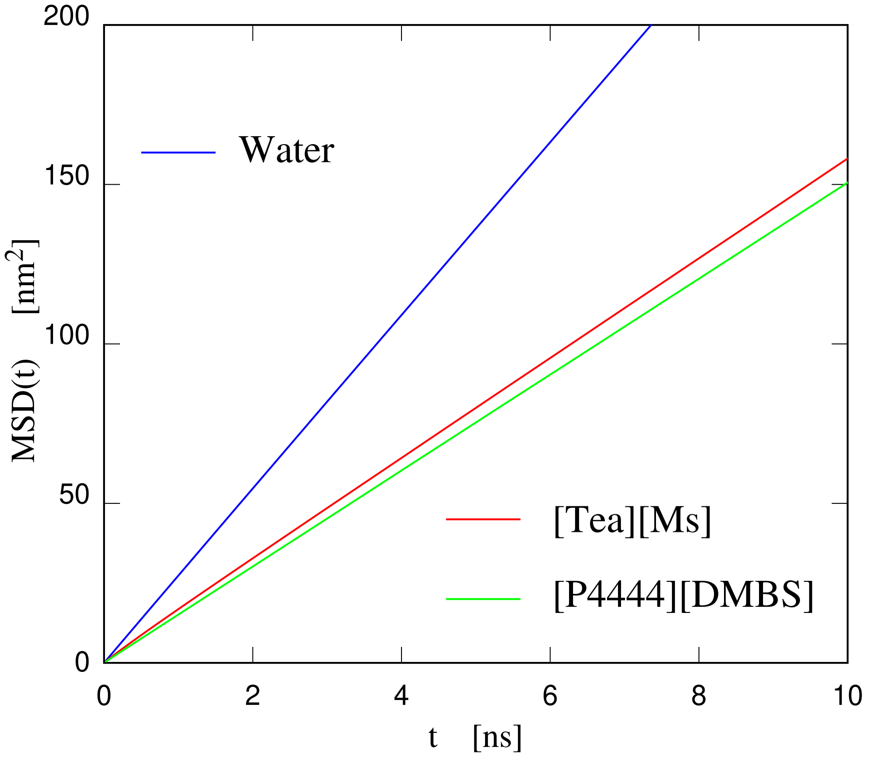

FIG. S16: Mean square displacement of water molecules as a function of time in pure water and in the two IL / water solutions at 25 wt% IL concentration. Homogeneous samples at $T = 300$ K and $P = 1$ atm. In the case of water, the diffusion constant estimated through the Einstein relation is equal to the SPC value reported in the literature, and, as such, it is somewhat overestimated. However, we rely on the computed $MSD(t)$ to estimate the ratio of viscosity in the three samples using the Stokes-Einstein relation as $\eta_\alpha/\eta_\beta = D_\beta/D_\alpha$ where η is the viscosity, D is the diffusion constant, and α, β refer to any two samples among the three we simulated. In this way, we estimate that the ratio of viscosity in water, [Tea][Ms] and [P_{4444}] is 1 : 1.72 : 1.79, respectively. These ratios are used to parametrise the Rayleigh-Plesset equation that provides a hydrodynamic description of the collapse of bubbles in the corresponding samples.

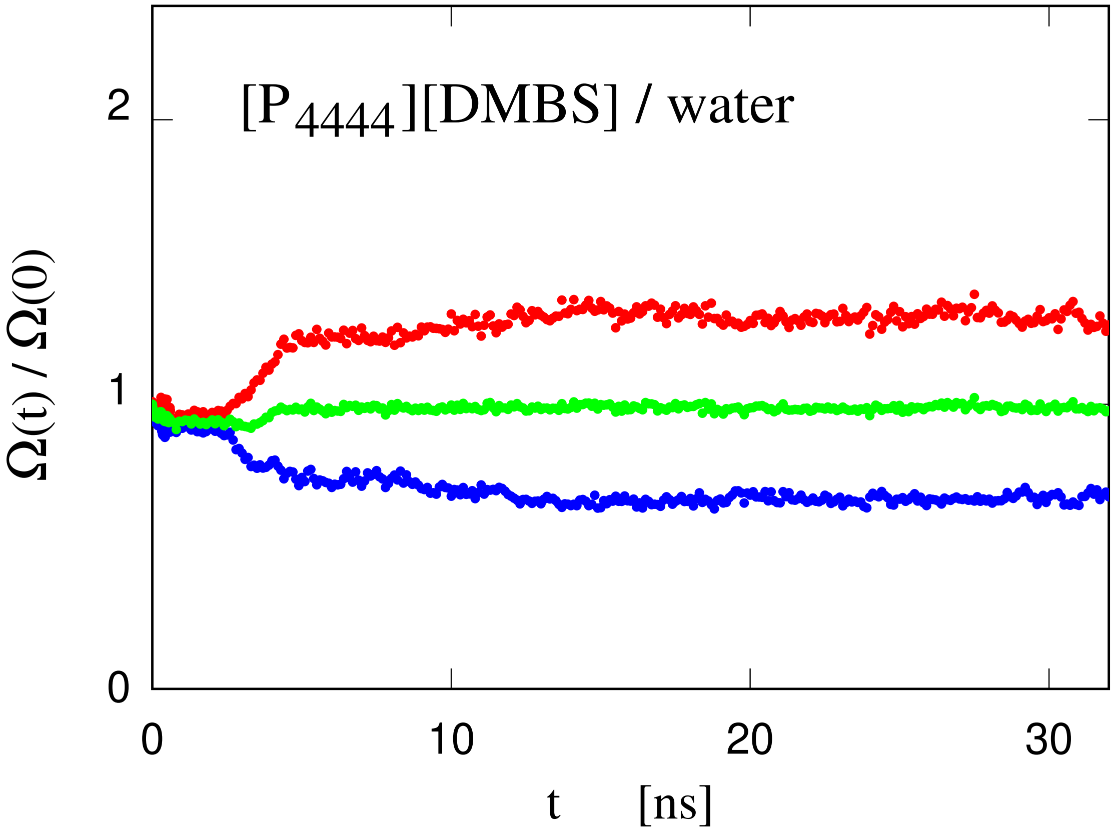

FIG. S17: Time dependence of the volume of two cavities in the [P_{4444}][DMBS] / water solution, initially of equal volume (see text). Red dots: growing cavity; blue dots: receding cavity; green dots: average of the two volumes. To ease comparison, all volumes have been scaled by the common volume of the two bubbles at the beginning of their evolution ($t = 0$). Moreover, the vertical scale [0 : 2] is the same of the pure water and [Tea][Ms] / water cases. It is apparent that the equilibration to the expected state consisting of a single bubble whose volume is the sum of the two initial volumes is exceedingly slow.

The nucleation and growth of cavities observed by simulation at NVT condtions

The observations on the nucleation and growth of cavities obtained through simulation at NVT conditions, can be rationalised in terms of the static Laplace equation (Eq. 5 of the main text). Let us consider a homogeneous liquid under tension θ_{start} at volume V, whose equilibrium volume at $P = 1$ bar is V_0. After its formation, the nanometric bubbles at equilibrium in the

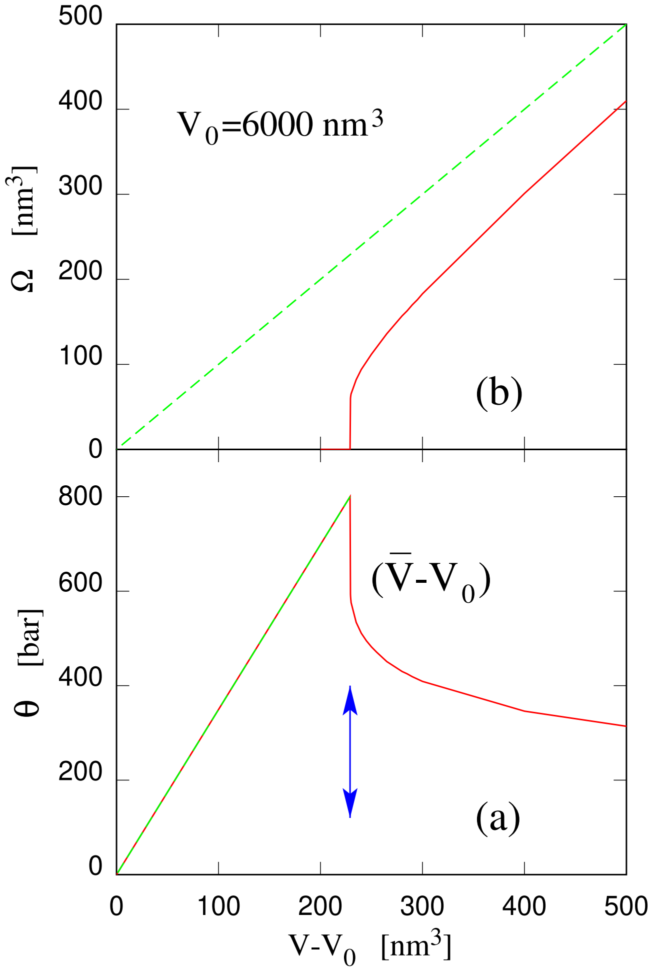

FIG. S18: Volume of the cavity and residual tension in a water sample of $V_0 = 6000$ nm^3 estimated by solving Eq. 3, assuming that the system evolves at NVT conditions. The straight line in panel (a) shows the pressure $P = -\theta = -B(V - V_0)/V_0$ in the homogeneous fluid under tension. The straight line in panel (b) shows the excess sample volume $(V - V_0)$ with respect to the $P = 1$ bar condition, which represents an upper bound for Ω. No cavity can form for $V < \bar{V}$.

equally mesoscopic samples contain a very dilute vapour corresponding to the water vapour tension at $T = 300$ K. This dilute vapour has positive but negligible pressure. Hence, to estimate the order of magnitude of the different parameters, we assume $p_{in} = 0$. Then, in the presence of the bubble, the residual pressure in the (no longer homogeneous) liquid phase is

(see Eq. 5 of the main text):

$$p_{out} = -\frac{2\gamma}{R} \tag{1}$$

where γ is the surface tension of water and $R = (3\Omega/4\pi)^{1/3}$ is the radius of the bubble.

After the cavity formation, the liquid will occupy a volume $(V - V_0 - \Omega) > V_0$, with a residual tension that can be estimated as $\theta_{res} \equiv \theta_{out} = B(V - V_0 - \Omega)/V_0$. Therefore, the volume Ω of the equilibrium bubble has to satisfy:

$$p_{in} = 0 = -\theta_{res} + \frac{2\gamma}{R} \tag{2}$$

or:

$$0 = -B\left(\frac{V - V_0 - \Omega}{V_0}\right) + 2\gamma\left(\frac{4\pi}{3\Omega}\right)^{1/3} \tag{3}$$

On the one hand, the volume Ω of the bubble cannot exceed $V - V_0$, otherwise the residual tension would be negative (i.e., $p_{out} > 0$), a condition hardly compatible with the presence of a stable cavity. On the other hand, because of the $2\gamma/R$ term, the cavity cannot be arbitrarly small otherwise this term could not be balanced in Eq. 3. Eq. 3 has been solved numerically as a function of volume V for a sample whose equilibrium size $V_0 = 6000$ nm^3 is comparable to that of the Large Water sample. The results, obtained using the experimental $\gamma = 0.072$ N/m and $B = 2.1$ GPa of water at $T = 300$ K, are shown in Fig. S18. More details are given in SI. Already from Fig. S18, it is apparent that, because of surface tension, for any given V_0 there is a minimum volume $\bar{V} > V_0$ and a corresponding minimum tension $\bar{\theta} = B(\bar{V} - V_0)$ under which no stable cavity could form. In the mesoscopic range ($\Omega \sim 10^3 - 10^4$ nm^3), the residual tension predicted by the model is sizeable, amounting to a few 10^2 bar. Moreover, above $\bar{V}$, the residual tension θ_{res} increases with reducing V, instead of tending monotonically to zero with $V \to V_0$. This somewhat counterintuitive behaviour (as pressure decreases with reducing volume) is due to the increasing weight of the water/vapour surface tension in the pressure balance of the system. Last but not least, the simple model represented by Eq. 3 shows how the cavity accounts for a fraction of the excess volume $(V - V_0)$ which increases with increasing V, as found by simulation.

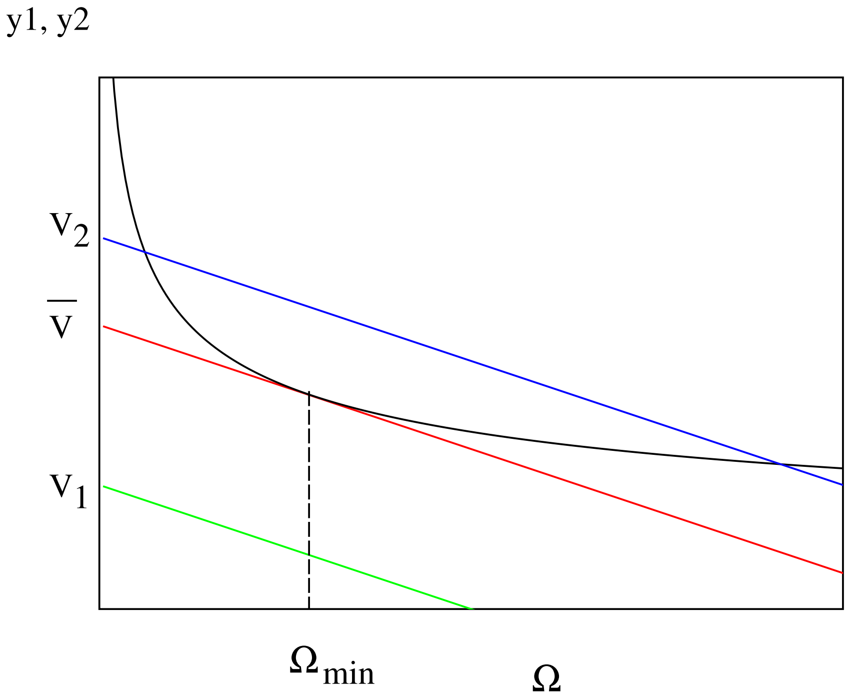

FIG. S19: Graphical solution of Eq. 8 of the main text. The condition stated by Eq. 8 that identifies the equilibrium volume Ω of a cavity in a volume $V > V_0$ (see text) can be solved graphically by crossing the curves $y1(\Omega) = B(V - V_0 - \Omega)/V_0$ and $y2(\Omega) = 2\gamma(4\pi/\Omega)^{1/3}$ for any given V, V_0. No $\Omega > 0$ solution is found for $V < \bar{V}$, volume at which a bubble appears with a non-zero volume Ω_{min}. At $V > \bar{V}$ the two curves cross at two Ω values, but it is immediate to verify that only the second is a minimum of the free energy. The first crossing is a maximum of free energy, and is reminiscent of the activated state for nucleation.

Needless to say, these considerations strictly depend on the NVT conditions of the simulation and of the model, and also on the mesoscopic size of the simulated sample. Nevertheless, they are useful to interpret the simulation data and could be relevant for cavitation in porous media or, in general, in confined geometries.

Considerations on the collapse of cavities in water based on the Reyleigh-Plesset equation

For what concerns the water case, the RP can therefore be used to make simple predictions that represent a benchmark for our investigation of IL effects. First, at zero pressure and considering (spherical) cavity sizes in the μm range (thus beyond the sizes considered in simulation), the time evolution is mainly determined by viscosity. Excluding the very initial and final stages of collapse, in which inertia is relevant, the RP equation can be approximated as:

$$\frac{\dot{\Omega}}{\Omega} = -\frac{3\gamma}{2\eta}\left(\frac{4\pi}{3\Omega}\right)^{1/3} \tag{4}$$

whose solution (with boundary conditions $\Omega(t) = \Omega_0$ for $t \leq 0$, and $\Omega(t) = 0$ for $t \geq \bar{T}$) is:

$$\Omega(t) = \left[\Omega_0^{1/3} - \frac{9\gamma}{2\eta}\left(\frac{4\pi}{3}\right)^{1/3} t\right]^{1/3} \qquad 0 \leq t \leq \bar{t} \tag{5}$$

where $\bar{t}$:

$$\bar{t} = \frac{2\eta}{9\gamma}\left(\frac{3\Omega_0}{4\pi}\right)^{1/3} \tag{6}$$

is the time required for the bubble to collapse. Since $\bar{t}$ grows linearly on the initial radius of the bubble, the velocity of the cavity surface during collapse will be virtually the same at all sizes, and no extreme heating will take place. However, for pressure p_{out} exceeding a few bar, the RP equation can be approximated as:

$$\frac{\dot{\Omega}}{\Omega} = \frac{3p_{out}}{4\eta} \tag{7}$$

Hence, the rate of volume reduction is proportional to the volume, and the velocity of the approaching surfaces will also grow linearly with the cavity size. A simple estimate using the viscosity of water and pressures in the $\sim 10^2$ range shows that for cavities of μm radius, this velocity will reach the sound velocity, generating the shock waves which play a crucial role in sonochemistry and sonoluminescence.
